# Postharvest Fruit Grading Technologies and Equipment: A Review

**DOI:** 10.3390/foods15183308

**Published:** 2026-09-18

**Authors:** Jianli Hu, Lixin Ma, Wenya Zhang, Jinxiu Song, Pengpeng Yu

**Affiliations:** 1 School of Agricultural Engineering, Jiangsu University, Zhenjiang 212013, China; 2 School of Food Science and Engineering, Jiangsu University, Zhenjiang 212013, China; 3 College of Water Resources and Intelligence Engineering, China Agricultural University, Beijing 100083, China

**Keywords:** Postharvest fruit grading, nondestructive quality assessment, data-processing algorithms, multisensor fusion, intelligent grading equipment

## Abstract

Postharvest fruit quality differs among various fruits and alters during sorting, packaging, transportation and storage. Therefore, it is important to have an efficient and objective grading method that causes little mechanical damage to ensure the uniformity of products and decrease market price and losses in the supply chain. This paper describes the grading norms, detection techniques, processing algorithms, structural designs and practical uses in different types of fruits. It examines the external features, internal quality and hidden faults by using machine vision, visible-near-infrared spectroscopy, hyperspectral and X-ray imaging, acoustic and mechanical detection, electronic noses and the integration of multiple sensors. In addition, it also investigates conventional machine learning, deep learning, transfer learning and lightweight implementations. The research has developed from grading according to size, weight and color to a complete evaluation of ripeness, juice volume, hardness, internal defects and shelf life. Moreover, single detection devices are joined together to construct integrated systems including feeding, separation, inspection, classification, redirection, packaging and data management. However, the application is restricted by the discrepancy between the grading criteria and measurable results, the lack of cross-species and batch generalization ability, and the difficulty in coordinating multiple sensors in real time. The systems should maintain a balance between production speed, mechanical damage and costs. Some matters needing attention are to standardize the quality description, choose multi-source fusion, develop adaptive lightweight models, design modular structures and guarantee end-to-end traceability. Solving these problems will facilitate the transition from accurate laboratory identification to reliable, economic and extensive commercial grading.

## 1. Introduction

Fruit is an important, highly perishable, and heterogeneous commodity in the global fresh-produce supply system. After being harvested, fruit continues to undergo metabolic activities such as respiration, transpiration, ripening and wilting. It is also affected by compression, impact, vibration and changes in temperature and humidity during sorting, packaging, transportation, storage and sales. These factors result in water loss, softening, mechanical damage and microbial deterioration, which decrease the marketable quantity and increase the waste of resources. Earlier research indicates that fruit and vegetable losses take place during the whole process of postharvest handling, distribution and consumption, and mechanical injury not only causes the immediate decline but also promotes the subsequent physiological deterioration and invasion by pathogens [1,2]. Therefore, it is necessary to carry out timely, objective and consistent sorting and grading according to measurable quality characteristics for the standardization of products, differentiated prices, suitable packaging and logistics, and reduction in supply chain losses.

The range of fruit grading has been extended from single aspects like diameter, weight and color to multi-dimensional quality features including surface flaws, maturity, soluble solids content (SSC), acidity, firmness, dry matter content, internal disorders and shelf life. Manual grading depends on visual and tactile judgment, but is influenced by the operator’s ability, fatigue, subjective standards and working conditions, which results in difficulty in making the same decisions during rapid operation. Although the mechanical systems based on the aperture size, roller spacing or weight can improve the production rate, they mainly sort the fruits according to a few physical parameters and cannot accurately detect hidden defects or the quality differences inside. Recently, systematic reviews show that research attention in fruit quality evaluation is focused on machine learning, deep learning and machine-vision systems, with the emphasis changing from the comparison of the classification accuracy offline to the detection of multiple attributes, real-time processing and adaptation to the industrial environment [3,4].

For external quality inspection, the machine-vision systems using RGB, multispectral and three-dimensional imaging can obtain information on fruit size, shape, color, texture and surface defects without any contact. The previous methods mainly depended on the threshold segmentation, color-space transformation and hand-crafted features and thus had poor robustness to background changes, variations in illumination, fruit orientation and defect size. Conversely, convolutional neural networks, object detectors and instance-segmentation models acquire hierarchical features from a large number of image data and enhance the recognition of various defects in different quality levels. Xu et al. installed an enhanced YOLOv5 model in an automatic apple-evaluating machine, connecting external-defect detection immediately to the actuator control [5]; Ji et al. integrated multi-angle image analysis with deep learning to improve the coverage of apple surfaces, suggesting that full presentation of the surface and fusion of multi-view features are crucial for decreasing the number of missed detections [6]. These advances have moved vision-based fruit inspection from single-view, single-feature decisions toward full-surface, multi-feature, and online grading.

External grades cannot fully characterize the eating quality or storage risk of fruit because visually sound fruit may still exhibit insufficient sweetness, reduced firmness, internal browning, insect infestation, or incipient decay. Nondestructive techniques, including visible–near-infrared spectroscopy, hyperspectral imaging, X-ray imaging, acoustic sensing, dielectric measurements, and electronic noses, have therefore been applied to evaluate chemical composition, tissue structure, maturity, and latent defects, increasingly in combination with chemometrics and intelligent algorithms [7]. The online near-infrared grading device developed by Wang et al. for fresh jujube demonstrated that spectral prediction models can be embedded in continuous conveying and grade-diversion operations [8]. Li et al. combined spectral-band selection with image processing to rapidly identify incipient decay in citrus fruit [9]. In general, a single sensor captures only a limited aspect of quality; multimodal sensing and data fusion are therefore important for the integrated grading of external and internal attributes.

Modern fruit-grading equipment is no longer an isolated inspection module but an electromechanical information system comprising feeding and cleaning, fruit singulation, orientation control, stable image or spectral acquisition, intelligent classification, sorting actuation, and data management. Chakraborty et al. integrated cleaning, vision-based sorting, weight grading, a lightweight deep network, embedded computing, and actuators into real-time citrus-grading equipment, confirming the need to co-design sensing models and mechanical structures [10]. In commercial fruit-handling operations, equipment performance depends not only on algorithmic accuracy but also on throughput, fruit rotation and occlusion, motion blur, illumination stability, sensor cost, sanitation and maintenance requirements, and control of mechanical damage. High accuracy under laboratory conditions therefore does not necessarily translate into stable grading performance on a production line.

Intelligent fruit grading still faces several common challenges. Biological variation associated with cultivar, production region, season, maturity, and storage conditions weakens model generalizability across batches. Visual similarity between defects and normal pigmentation, spatial heterogeneity in internal quality, and signal fluctuations during high-speed motion increase the risk of misclassification. Although multisensor systems provide complementary information, they also introduce difficulties in data synchronization, model complexity, equipment cost, and online computational demands [4,7]. Moreover, many studies continue to emphasize recognition performance on laboratory datasets while paying insufficient attention to commercial-grade definitions, reliable continuous operation, flexible low-damage handling, model calibration and maintenance, and life-cycle economics.

Based on the above situation, this paper investigates the fruit-grading standards, main nondestructive sensing techniques, intelligent classification procedures, grading devices and the system structure, as well as their applications to typical fruit types. Special emphasis is placed on the technical features and practical limits of machine vision, near-infrared spectrometry, hyperspectral imaging, other physical sensing means and the integration of multiple sensors in the online grading process. The interaction between the sensing models, transportation systems, orientation adjustments, control mechanisms and the operating devices for sorting is also examined, together with the issues concerning the extension of the models, light-weight installation, compliance with grading specifications, minimal damage during operation and data-driven management. Recent reviews have generally focused on a single sensing modality or algorithm family [3,4]. This review examines fruit grading across biological quality formation, sensor evidence, algorithm validation, and integration into grading equipment and commercial production lines. The assessment covers predictive accuracy and whether the complete system can maintain reliable grading across operating conditions while limiting fruit damage at an economically viable cost.

### Review Methodology

This article uses a structured narrative-review design because the topic spans biological quality, sensing, algorithms, machinery and commercial deployment. The 123 references were identified through keyword searches in the Web of Science database. The coverage period was 1 January 2017 to 30 August 2026. Search terms combined (“postharvest” OR “post-harvest”) AND fruit AND (grading OR sorting OR “quality assessment”) with modality terms (machine vision, Vis/NIR, hyperspectral, X-ray, acoustic, electronic nose and sensor fusion) and equipment terms (conveyor, actuator, packing line, online and industrial).

Studies were retained when they addressed postharvest fruit quality or grading and reported a measurable sensing, algorithmic, mechanical, validation or deployment outcome. Preharvest-only studies, unrelated commodities and reports without direct engineering relevance were excluded; contextual engineering sources were retained only when their mechanisms informed sensing or handling design. Representative empirical studies selected for Table 1 had to cover a distinct sensing modality, algorithm, equipment configuration or fruit-handling context, report at least one quantitative performance outcome and meet at least one prespecified scale or operational benchmark: at least 200 individual fruit or fruit clusters, at least 2000 labeled images or fruit instances, at least 4 fruit s − 1 or at least 25 frames s − 1. Studies below these thresholds remained eligible when they included independent validation across batches, cultivars, seasons or sites, or reported an online, pilot-line or commercial-line trial. These cutoffs were used as transparent selection benchmarks for this heterogeneous narrative review rather than as universal definitions of adequate study size or industrial throughput.

Evidence maturity was scored from 0 to 2 in six domains: sample scale, biological and environmental diversity, validation independence, operational setting, throughput, and reproducibility/reporting (Supplementary Methods S1 and Table S1). NR received no credit, and borderline values were assigned conservatively to the lower category. Coding differences were resolved by source rechecking; unresolved cases received the lower score. Overall categories controlled synthesis weight and included validation and deployment gates, so sample scale or speed alone could not support transferability or commercial-readiness claims. Table S2 reports item-level scores for Table 1 studies, and Table S3 maps evidence roles for all 123 references (Supplementary Materials). Because the objective was integrative rather than meta-analytic, no pooled effect estimate was calculated.

**Figure 1:**
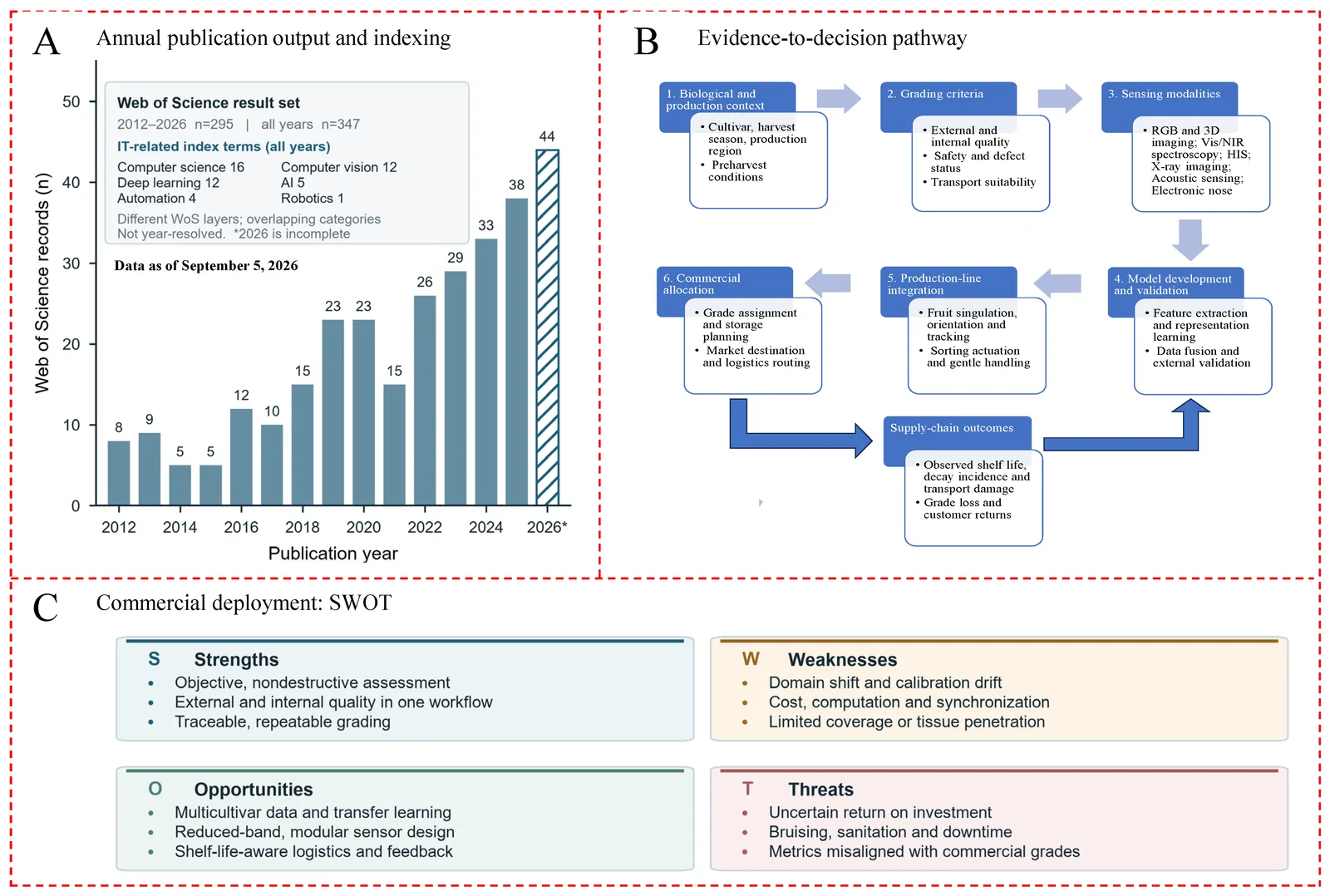
Integrative synthesis of postharvest fruit-grading research. (**A**) Annual publication counts based on a Web of Science “Analyze Results” export for the search phrase “postharvest fruit grading”. The export contained 347 records in total, 295 of which were published between 2012 and 2026, the 15-year period shown in panel (**A**). The 2026 count is incomplete. The inset gives technology-related indexing counts for all 347 records. These categories overlap, and the export does not break down their counts by publication year. (**B**) Evidence-to-decision architecture linking biological and production context, grading criteria, sensing modalities, model development and validation, production-line integration, commercial allocation and outcome feedback. (**C**) SWOT synthesis of commercial postharvest fruit-grading systems. The figure is original to this review.

A Web of Science “Analyze Results” export for the search phrase “postharvest fruit grading” identified 295 records published between 2012 and 2026. Annual publication counts varied through 2021, then rose each year from 26 in 2022 to 44 at the time of the 2026 export. The full dataset included records indexed under computer science, computer vision and graphics, deep learning for image recognition, artificial intelligence, automation and control systems, and robotics. These categories show that the field overlaps with several areas of information technology. However, because the export summarizes publication years and topics separately, it cannot link the annual increase to any specific technology. The 2026 count covers only part of the year (Figure 1A).

## 2. Quality Criteria for Postharvest Fruit Grading

Postharvest fruit grading should consider commercial appearance, eating quality, safety, and suitability for distribution. External traits include size, mass, shape, color, surface cleanliness, and visible defects. Internal traits include soluble solids content (SSC), acidity, sugar-to-acid ratio, firmness, dry matter, moisture, and maturity. Safety and defect assessment cover internal browning, hollow tissues, chilling or freezing injury, insect damage, mold, and decay, while distribution suitability depends on storability, shelf life, maturity uniformity, and resistance to transport damage. Together, these factors determine commercial grade, eating value, and supply-chain allocation. Kyriacou and Rouphael described fresh fruit quality in terms of appearance, sensory, nutritional, functional, and safety attributes. Grading systems should therefore move beyond size or mass alone and assess quality across several dimensions [22]. The measured phenotype is also conditioned by genotype, crop load, developmental stage and the preharvest environment; these factors can alter composition, firmness, maturity and storage potential before fruit reaches the grading line [22]. A grading model should therefore be interpreted within the production contexts represented in its calibration and validation data.

External attributes are easy to observe, measure quickly, and incorporate into online systems, so they remain central to commercial grading. Weight sensors and two-dimensional or three-dimensional vision are commonly used to assess fruit diameter, shape, color, and surface defects. Internal attributes are more closely related to flavor, texture, and maturity, but conventional physicochemical tests are destructive and limited to sample inspection. Visible and near-infrared spectroscopy enables rapid, nondestructive estimation of soluble solids content (SSC), dry matter, firmness, and maturity. Walsh et al. reviewed point measurements using this technique for harvest timing and commercial assessment of internal fruit and vegetable quality, providing a basis for moving internal quality evaluation from laboratory sampling to online grading [23]. External traits are therefore suited to rapid initial sorting, whereas internal traits support finer grading and pricing based on quality, particularly for valuable fruit.

Safety and defect indicators should be assessed first. Surface lesions, cracks, and advanced decay can usually be detected visually, but internal browning, watercore, cavities, latent decay, and early mechanical injury may show no external signs. Detecting these conditions requires spectroscopy, X-ray imaging, acoustic sensing, nuclear magnetic resonance, or multisensor fusion. Qiao et al. combined hyperspectral imaging with low-field nuclear magnetic resonance to identify decayed blueberries nondestructively. Their findings indicate that combining spatial, spectral, and tissue water information improves the reliability of latent defect classification [24]. Such defects should not be treated as ordinary differences in grade. Fruit associated with decay, disease, or consumption risk should be rejected before the remaining fruit are assigned commercial grades.

Distribution suitability refers to the ability of fruit to retain quality during packaging, storage, transport, and sale. Its assessment considers maturity, firmness, shelf life, resistance to impact and vibration, and the risks of water loss and decay. Impact, compression, and vibration during transport can cause bruising and cracking, disrupt internal tissues, accelerate physiological deterioration, and promote microbial invasion. Al-Dairi et al. systematically examined these effects and emphasized that cushioning, loading configurations, and logistics conditions should match the fruit’s susceptibility to damage [25]. A practical grading sequence is therefore to reject fruit with safety-related defects, sort the remaining fruit by external quality, refine the assessment using internal attributes, and allocate the resulting grades according to distribution performance. Distribution suitability should be expressed as an allocation decision—such as local sale, long-distance transport or extended storage—linked to predicted remaining shelf life, market destination and expected loss reduction rather than as a generic quality label. This framework provides a common basis for selecting sensing technologies, designing equipment, and making packaging, storage, and transportation decisions.

## 3. Key Technologies for Postharvest Fruit Grading

### 3.1. Machine Vision

Machine vision obtains information about the fruit diameter, shape, color, texture and surface defects from RGB, multiview or structured-light images. The main advantages are the high inspection speed, the low cost, the noncontact operation and the easy integration with the conveying and rejecting devices, which makes it suitable for high-speed external screening and online grading. The conventional thresholding, edge-based and texture-based algorithms are easily understandable and suitable for a single task under steady light and background. While Convolutional Neural Networks (CNNs), YOLO detectors and instance-segmentation models are more competent to deal with complicated defects, different fruit positions and small targets, they need representative annotated data sets and should eliminate the influences of specular reflection, occlusion, motion blur and cross-batch domain variation.

**Figure 2:**
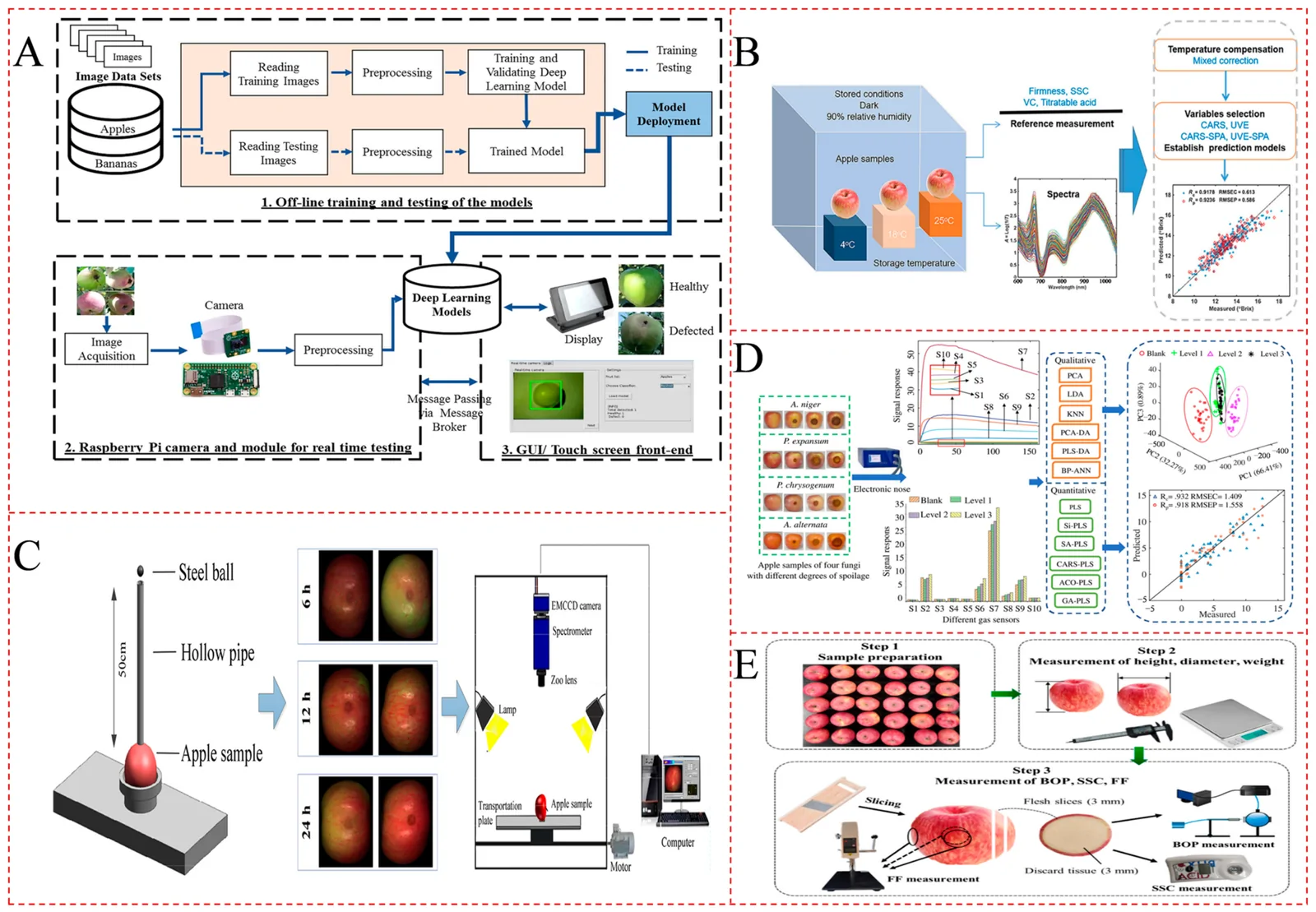
Representative sensing technologies for postharvest fruit grading. (**A**) Real-time computer-vision and deep-learning system for fruit grading [26]. (**B**) Experimental workflow for collecting apple NIR transmittance spectra at different temperatures and developing reference-based quality models [28]. (**C**) Hyperspectral image-acquisition system [29]. (**D**) Gas-sensor workflow for identifying spoilage fungi and estimating spoilage severity in apples [30]. (**E**) Measurement cycle for optical-property and quality assessment; the dashed circle in Step 3 indicates sampling on the reverse side of the apple [31]. SSC, soluble solids content; FF, fruit firmness; BOP, bulk optical properties.

Ismail et al. developed a real-time vision system using EfficientNet and Raspberry Pi for fruit classification, such as apples and bananas. Test-set accuracies were 99.2% and 98.6%, respectively, whereas online accuracies for real samples were 96.7% and 93.8%, indicating that low-cost edge devices can support rapid external grading on standardized packaging lines [26] (Figure 2A). Chen et al. employed a lightweight YOLO-BFD model to classify bananas into four freshness grades, achieving a mean accuracy of 97.8% and detection speeds of 61 and 29 frames/s on the Jetson Orin NX and Jetson TX2, respectively. The system is therefore suitable for portable real-time inspection in retail, storage, or small-scale sorting environments [27].

Further studies show that conventional RGB and multiview imaging have expanded from color and surface-defect assessment to fruit-shape symmetry, cluster compactness, quantitative maturity assessment, and cultivar identification [32,33,34,35,36,37]. Enhanced imaging based on structured-light reflectance can further reveal incipient decay and shallow latent defects that are difficult to visualize using conventional RGB imaging [38,39]. Recent reviews indicate that the principal challenge in online machine-vision grading has shifted from maximizing accuracy on a single dataset to balancing consistency with grading standards, cross-cultivar transferability, computational requirements, and production-line throughput [40]. Training and external-test data should therefore span peel morphology, natural pigmentation, defect prevalence, background, illumination, fruit orientation and conveyor speed. A model should not be described as transferable until it has been tested on an unseen cultivar, site, season or production batch. Studies should report not only accuracy, precision, recall, and mean average precision (mAP), but also independent-batch performance, processing time per fruit, model size, and misclassification categories. Production-line applicability should be further improved through multi-view coverage, illumination correction, and lightweight deployment (Table 2).

### 3.2. Near-Infrared Spectroscopy

Visible–near-infrared (Vis/NIR) spectroscopy estimates soluble solids content (SSC), dry matter content, firmness, and maturity from the absorption and scattering responses of functional groups such as O–H and C–H. The technique is nondestructive, enables rapid single-sample measurements, and can be implemented in either handheld instruments or online grading systems, making it particularly suitable for internal-quality classification of high-value fruit. Its principal limitations are that measurements generally represent localized point information and that model performance is readily affected by cultivar, origin, season, temperature, peel characteristics, and measurement position.

Minas et al. used a single near-infrared scan to predict the internal quality of peaches. The dry matter and SSC models achieved R^2^ values of 0.98 and 0.96 and RMSEP values of 0.41% and 0.58%, respectively, supporting the application of this technique to the postharvest selection of premium fruit and maturity management [44]. Fan et al. developed a portable 400–1000 nm device for apples and predicted SSC using dynamic correction with white-reference, dark-reference, and sample spectra. Independent laboratory validation yielded an R^2^ of 0.764 and an RMSEP of 0.672%; after recalibration using field data, the corresponding values were 0.690 and 0.604%, respectively. These results also demonstrate that portable instruments require environment- and batch-specific calibration for use under field conditions [11].

The engineering reliability of Vis/NIR models depends primarily on adequate coverage of sample variability and rigorous control of measurement conditions. Studies involving general models for apples from different production regions, dynamic quality monitoring under different storage temperatures, and simultaneous detection of watercore and SSC indicate that calibration sets should represent variation in origin, temperature, storage stage, and internal-defect status. Model stability should be maintained through independent validation across multiple batches, temperature compensation, instrument-to-instrument calibration transfer and versioned model updating [28,45,46] (Figure 2B). Routine commercial use also requires reference-standard checks, optical-window cleaning and drift monitoring. Vis/NIR spectroscopy is therefore suitable for refined internal-quality grading, but evidence beyond a high R^2^ from a single randomly partitioned dataset is needed to establish online applicability (Table 2).

### 3.3. Hyperspectral Imaging

Hyperspectral imaging records a continuous spectrum at each pixel, combining spatial localization with compositional response. It can reveal early bruising, decay, and disease that are difficult to discern in RGB images and can map the spatial distribution of defects or quality attributes. This information density makes the technique suitable for latent-defect screening of premium fruit, offline precision inspection, and the identification of informative wavelengths; however, equipment cost, calibration requirements, and data dimensionality are relatively high. For engineering deployment, a small number of characteristic wavelengths are typically selected and transferred to multispectral cameras and lightweight models to balance inspection speed and cost.

Tian et al. acquired visible–near-infrared hyperspectral images of apples at 6, 12, and 24 h after impact. Following two-band ratio processing and improved watershed segmentation, recognition rates for sound fruit, bruised fruit, and all samples were 93.3%, 92.2%, and 92.5%, respectively. The study further reduced the feature set to two bands, supporting subsequent online multispectral implementation [29] (Figure 2C). Chun et al. combined hyperspectral fluorescence imaging with a ResNet-50-based one-dimensional convolutional neural network (1D-CNN) to distinguish healthy, asymptomatic, infected, and post-infection strawberries. The model achieved an accuracy of 96.86%, with precision, recall, and F1-score values of approximately 96.9%, demonstrating its suitability for the early warning of gray mold during packaging and distribution [47].

Further studies indicate that quality-related deep-feature learning, compensation for fruit-scale variation, and reconstruction using selected wavelengths can improve the representation of internal quality and weakly visible defects in hyperspectral data while facilitating the transition from full-spectrum experimental systems to few-band detection devices [48,49,50]. Hyperspectral models should therefore be evaluated not only in terms of classification accuracy or regression error but also for their stability across cultivars, batches, and imaging conditions. The number of selected wavelengths, processing time per fruit, calibration and maintenance burden, hardware cost and expected value recovery should be assessed concurrently. In most current settings, full hyperspectral imaging is more defensible for high-value fruit, offline audits or wavelength discovery than as an unqualified replacement for RGB or Vis/NIR sensing on a main commercial line (Table 2).

### 3.4. X-Ray, Acoustic, Mechanical, and Electronic-Nose Techniques

X-ray imaging exploits differences in tissue density and is suitable for identifying externally invisible defects such as internal browning, cavities, and insect infestation. Acoustic and mechanical methods evaluate firmness, crispness, and maturity from tapping, vibration, compression, or puncture responses and generally require relatively simple hardware. Electronic noses monitor volatile organic compound fingerprints associated with maturity, injury, and decay and are more appropriate for storage-related early warning. These three categories serve distinct applications: X-ray imaging supports high-value internal inspection or quarantine; acoustic and mechanical sensing is suitable for texture grading; and electronic noses are appropriate for batch- or storage-area monitoring. Their shared constraints include equipment safety and cost, excitation consistency, environmental noise, and sensor drift.

Tempelaere et al. trained BraeNet using X-ray computed tomography (CT) and single two-dimensional radiographs to identify internal browning in ‘Braeburn’ apples induced by low-oxygen or high-CO_2_ storage. Both the two- and three-dimensional models achieved accuracies of 96% ± 1%, indicating that faster and less expensive two-dimensional imaging may replace full CT for this task [41]. Zhang et al. obtained kiwifruit vibration responses using a 6 cm free-fall impact and employed a PCA-BPNN model to classify fruit into three maturity stages: unripe, ripe, and overripe. Calibration- and validation-set accuracies were 94.2% and 92.1%, respectively. These results indicate that noncontact impact testing is suitable for maturity grading; however, the correlations of the measurements with SSC and acidity were weaker than those with firmness and sensory attributes, indicating that the intended assessment target should be clearly defined [42].

Further studies indicate that combining X-ray CT with impact testing and image processing can advance internal-damage assessment from qualitative identification to the quantitative measurement of bruise volume and susceptibility to bruising. Electronic noses combined with pattern recognition or chemometrics can discriminate spoilage fungi and provide continuous early warning in storage environments [30,51,52] (Figure 2D). X-ray imaging is therefore best suited to high-value precision inspection of individual fruit, acoustic and mechanical sensing to rapid texture grading on conveying lines, and electronic noses to batch- or storage-area trend monitoring. Engineering applications must also control radiation exposure, excitation repeatability, temperature and humidity, and sensor drift. High accuracy under laboratory conditions should not be regarded as a substitute for validation in actual production environments (Table 2).

### 3.5. Multisensor Fusion

Multisensor fusion combines visual, spectral, acoustic, mechanical, or olfactory information at the data, feature, or decision level, enabling the simultaneous assessment of appearance, internal composition, and tissue status. It is therefore a key route from single-attribute detection to comprehensive quality grading. Data-level fusion retains the greatest amount of information but produces high-dimensional inputs; feature-level fusion balances predictive performance and computational demand; and decision-level fusion facilitates modular maintenance. The approach is most suitable for high-value fruit, multi-attribute pricing, and shelf-life prediction but requires solutions for synchronized acquisition, spatial registration, differences in measurement scales, missing data, and increased system cost.

Chen et al. fused acoustic-image features with visible–near-infrared spectra and employed SwinT-PLS to predict the firmness of yellow-fleshed peaches. The prediction set yielded R^2^ = 0.951, RMSEP = 0.443 N/mm, and RPD = 4.339, substantially outperforming the individual acoustic and spectral models and demonstrating suitability for commercial grading in which both processing speed and internal texture are important [43]. Fathizadeh et al. used the dominant acoustic frequency, dominant vibration frequency, and mass of apples as inputs to an artificial neural network and subsequently performed decision fusion. Feature-level fusion increased classification accuracy by an average of 10.84% and 10.14% for the two sample groups, whereas Dempster–Shafer decision fusion yielded maximum improvements of 19.8% and 12.5%, respectively. These findings indicate the value of complementary signals for shelf-life grading, although the benefits of fusion require confirmation across independent batches and under online operating conditions [53].

Recent studies show that multisource fusion has progressed from offline feature concatenation to the joint sensing of temperature, humidity, vibration, gases, and other environmental variables, together with cloud–edge collaborative inference. Comparisons of different optical representations acquired during the same measurement also indicate that additional modalities or features do not necessarily improve accuracy [31,54,55] (Figure 2E). Engineering systems should therefore quantify fusion gains against single-sensor baselines through ablation experiments and independent-batch validation. Synchronization error, robustness to missing modalities, inference time, and unit cost and maintenance burden should be reported concurrently to avoid systems in which additional sensors increase complexity without delivering commensurate benefits (Table 2). Across modalities, commercialization favors a tiered architecture: low-cost RGB for universal screening; Vis/NIR or reduced-band imaging for internal-quality or ambiguous cases; and X-ray, full hyperspectral imaging or multisensor fusion only when recovered product value justifies the added capital, calibration, computation and maintenance.

## 4. Data-Processing Algorithms for Fruit Grading

### 4.1. Conventional Machine-Learning Methods

Conventional machine learning uses manually extracted or selected structured features as inputs and primarily includes principal component analysis (PCA) for dimensionality reduction, partial least squares and partial least squares discriminant analysis (PLS/PLS-DA) for regression and classification, K-means clustering, support vector machines and support vector regression (SVM/SVR), random forests, and K-nearest neighbors (KNN). These models generally have fewer parameters, can be trained on smaller datasets, and offer greater interpretability. They are suitable for low-dimensional variables such as color, shape, texture, selected wavelengths, and acoustic parameters and often serve as benchmarks for small-sample fruit grading, quantitative spectral prediction, and newly developed algorithms. Their principal limitation is their dependence on feature engineering; renewed feature selection or model calibration is usually required when illumination changes, defects are complex, or data vary across cultivars (Table 3).

Yu et al. extracted fruit diameter, roundness, coloration ratio, and defect regions from top, bottom, and side images of apples and used weighted K-means clustering to assign four grades. The clustering-based grading accuracy was 93.3%, the integrated multi-feature grading accuracy exceeded 96%, and the processing speed reached 21 images/s, supporting its use in online grading under stable acquisition conditions and clearly defined grading rules [58]. Zhu and Spachos developed a mobile banana-grading system that cascaded an SVM with YOLOv3. In the first stage, the SVM classified ripeness from color and texture with an accuracy of 98.5%; in the second stage, YOLOv3 localized surface defects with an accuracy of 85.7%. This combination exploits the low computational cost of conventional classifiers, although defect-detection performance remains dependent on annotated samples and imaging conditions [62].

Further studies indicate that improvements in conventional algorithms have largely arisen from informative feature selection, preprocessing combinations, and parameter optimization. CARS-WOA-SVM has been used for goji-berry cultivar discrimination. SVM, LS-SVM, and hybrid models have been applied to dried-fruit quality assessment and adulteration detection. Near-infrared features combined with classifiers have enabled the identification of early injury in kiwifruit [56,59,63,64]. Random forests achieved a test-set accuracy of 93.10% for soft X-ray texture-based grading of internal blackheart in pomegranates and accuracies of 92.9–100% for optical-feature classification across cultivars of pear, apple, nectarine, and other fruits [60,61]. Conventional machine learning is therefore most appropriate when sample size is limited, feature dimensionality is manageable, and interpretable decision variables are required. Independent-batch or external validation should be performed, and the number of features, cross-validation strategy, test-set size, and inference time should be reported to prevent model performance from being overestimated because of small-sample partitioning or feature leakage.

### 4.2. Deep-Learning Methods

Deep learning automatically learns features from images or one-dimensional spectra [57,65,66,67]. Common fruit-grading tasks include whole-fruit grade classification, object and defect detection, and pixel-level segmentation. CNNs are suitable for maturity-stage or sound/defective classification; one-stage models such as YOLO and SSD provide rapid inference and are well suited to online localization; Faster R-CNN generally offers high detection accuracy but requires greater computational resources; U-Net, DeepLab, and BiSeNet can quantify defect areas; and Transformers are effective at modeling global relationships but typically require larger datasets and greater computing capacity. Algorithm selection should be determined by the required output: classification networks are appropriate when only grade labels are needed, whereas detection and segmentation networks should be used when defect locations and areas, respectively, must be determined (Table 4).

Arunima et al. developed a CNN-based maturity model for Nendran bananas using 4320 images and classified the fruit into four stages: unripe, partially ripe, ripe, and overripe. Compared with VGG16, VGG19, InceptionV3, ResNet50, and EfficientNetB0, the proposed model achieved an accuracy of 95% and was subsequently deployed as a web application for rapid postharvest maturity grading [68]. Liang et al. combined BiSeNet V2 semantic segmentation with a pruned YOLOv4 model to grade apples online according to the number and actual area of defects. BiSeNet V2 achieved a mean pixel accuracy of 99.66% and an inference time of 9 ms per image, while the complete system achieved an online grading accuracy of 92.42% and an F1-score of 94.31%. These results indicate that a segmentation-plus-detection-correction strategy is well suited to refined defect grading of high-value fruit [12].

### 4.3. Model Transfer, Lightweight Design, and Online Deployment

Model transfer, lightweight design, and online deployment aim to preserve stability and efficiency when algorithms move from laboratory datasets to continuous grading lines. Transfer learning and domain adaptation use a limited number of target-domain samples to correct differences associated with cultivar, season, instrument, or operating environment [70], while spectral models commonly employ calibration transfer and model updating. Pruning, quantization, lightweight backbone networks, and knowledge distillation can reduce parameter count, storage requirements, and inference latency [71,72]. These algorithms should not be evaluated solely on accuracy; cross-batch performance, model size, floating-point operations (FLOPs), edge-device frame rate, and actual conveying capacity should also be reported (Table 5).

Pan et al. addressed seasonal performance degradation in visible–near-infrared apple-maturity models through model fusion and correction. For samples collected in 2020 and 2021, the corrected models increased the correlation coefficients by 6.8% and 10.6% and reduced the RMSE by 52.2% and 32.2%, respectively. These results show that seasonal correction is critical to the long-term use of spectral models and cannot be replaced by accuracy from a single test [73]. Sun et al. developed the G-YOLO-NK passion-fruit detection model using a GhostNet backbone, feature fusion, and knowledge distillation. The model achieved a mean average precision of 96.4%, occupied only 7.14 MB, and processed 11.23 frames/s on a Jetson Nano, representing a 50.4% reduction in model size relative to YOLOv5s. This study shows that edge deployment requires the joint optimization of accuracy, speed, and resource consumption [69]. Across algorithm families, maturity is determined by the evidence regime: conventional models remain competitive for small structured datasets, deep models are justified when annotated diversity and localization demands are high, and transfer or lightweight methods become decisive only when performance is demonstrated on unseen batches and target hardware.

## 5. Intelligent Postharvest Fruit-Grading Equipment and System Architecture

Intelligent postharvest fruit-grading equipment is not a stand-alone inspection instrument but an integrated electromechanical system in which material handling, quality sensing, data processing, and actuator control operate as a coordinated whole. As shown in Figure 1B, a typical process comprises the biological and production context, grading criteria, sensing modalities, model development and validation, production-line integration, commercial allocation and outcome feedback. Within the equipment layer, the front end determines whether the fruit is clean, orderly, and handled with minimal damage; the intermediate stage determines whether information is acquired completely and consistently; and the downstream stage converts grade assignments into accurate diversion actions. In addition to grading accuracy, system evaluation should consider throughput, fruit-surface coverage, control latency, mechanical damage rate, adaptability to cultivar changes, and maintenance cost [5,6,13,74].

### 5.1. Feeding and Cleaning Unit

**Figure 3:**
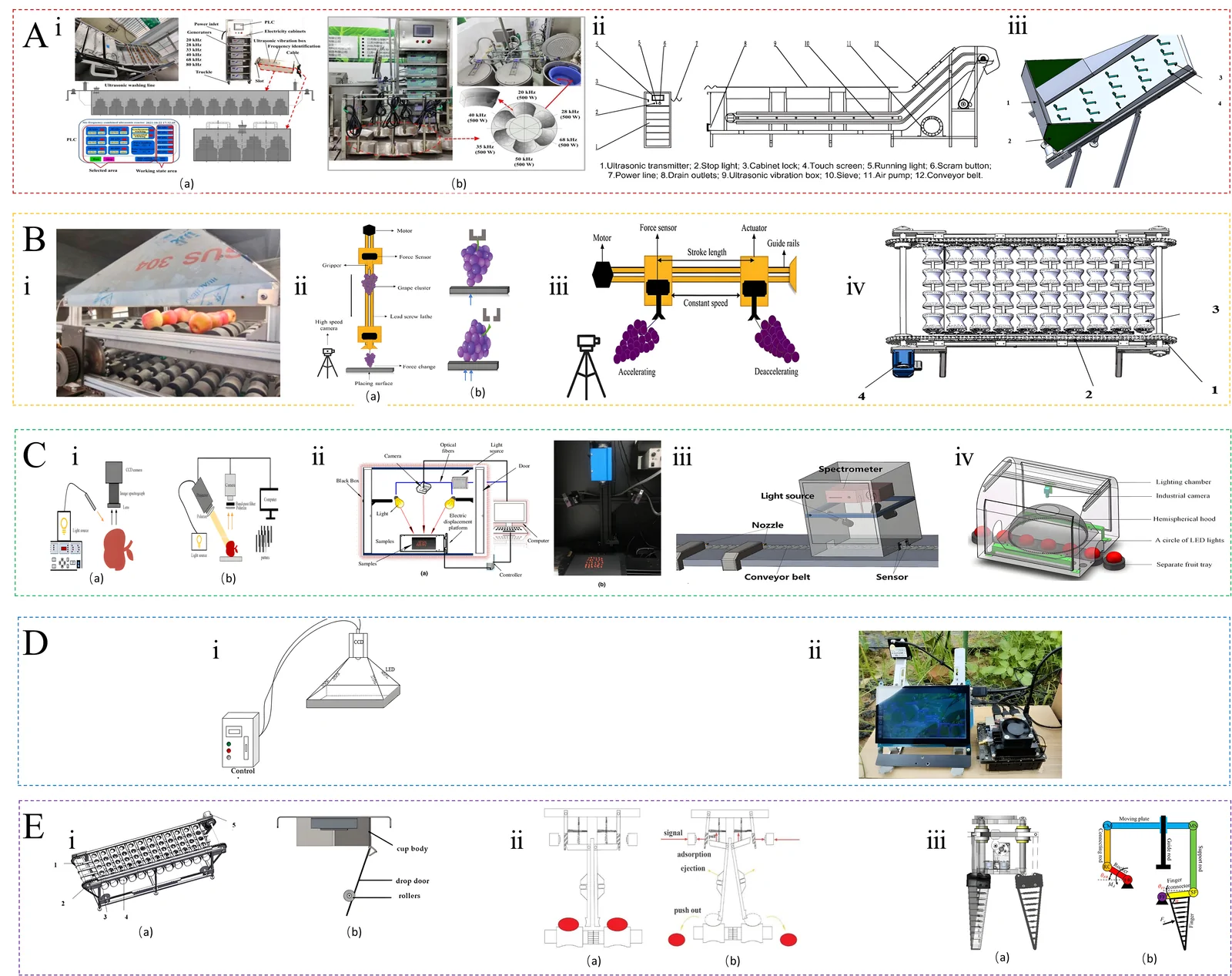
(**A**) Feeding and cleaning unit (**i**) (**a**) Multi-frequency power ultrasonic conveyor belt cleaning equipment. (**b**) Multi-frequency power ultrasonic centrifugal cleaning equipment [75], (**ii**) Structural diagram of ultrasonic cleaning machine [76], (**iii**) Turnover detection conveyor (1—sprockets, 2—chains, 3—sponge rollers, 4—motor) [5]. (**B**) Single-fruit conveying and posture adjustment unit (**i**) Roller turnover detection [6], (**ii**) Vertical start-and-stop conveying cycle: (**a**) vertical transportation; (**b**) placing view (The blue arrows indicate mechanical excitation from the placing surface during grape-cluster placement) [78], (**iii**) Schematic diagram of experimental observation of cluster’s vibration at acceleration and deceleration of actuator [79], (**iv**) Turnover detection conveyor. (1—sprockets, 2—chains, 3—sponge rollers, 4—motor) [5]. (**C**) Online detection unit (**i**) (**a**) Schematic illustrations of noncontact SRS systems; (**b**) schematic of an SFDI system for spectral image acquisition [77], (**ii**) (**a**), (**b**) Hyperspectral imaging system [63], (**iii**) Schematic of the grading-device hardware [8], (**iv**) Fruit surface area captured by the camera during rotation [14]. (**D**) Intelligent discrimination and control unit (**i**) Architecture of the visual inspection and automatic grading control system [5], (**ii**) Real-time detection environment using a Jetson Nano [69]. (**E**) Sorting actuation unit (**i**) Grading actuators. (**a**) Grading actuator; (**b**) Detail of grading fruit cup (1—Trigger grading mechanism; 2—Sprocket chain drive; 3—Grading fruit cup; 4—Grading channel; 5—Motor) [5], (**ii**) Schematic of actuator operation. (**a**) Non-working state; (**b**) Working state [15], (**iii**) Motion schematic of the gripper: (**a**) physical model; (**b**) kinematics model [80].

The feeding and cleaning unit performs three primary functions: continuous fruit supply, removal of foreign matter, and standardization of fruit-surface conditions. Common components include hoppers and elevator belts, cushioning water tanks, spray manifolds, brush rollers, ultrasonic cleaning tanks, drainage sections, and air knives [75]. Round, impact-tolerant fruit such as apples and citrus fruit can be processed using combined brush-roller and spray cleaning, whereas peaches, kiwifruit, and small berries are better suited to low-drop water flumes or flexible conveyors. The unit should be designed to prevent bridging, stacking, and high-speed drops while controlling cleaning pressure, brush-roller stiffness, and drying-air temperature to avoid abrasion or moisture loss caused by pretreatment [2] (Figure 3A) (Table 6).

Differences in soil contamination, water films, and wax layers on the fruit surface alter image reflectance and spectral responses; cleaning performance should therefore be evaluated alongside downstream inspection conditions. Zhou et al. summarized the principal components of ultrasonic cleaning systems, including spraying or immersion units, transducers, generators, and recirculating filtration systems. Cavitation enhances the removal of microorganisms and residues, but excessive power may generate noise, accelerate equipment erosion, and damage fruit tissues [76]. In engineering applications, cleanliness, damage rate, and residual moisture should be treated as joint evaluation criteria, and adjustable spraying, brushing, and air-drying modules should be incorporated.

### 5.2. Fruit Singulation, Conveying, and Orientation Unit

The fruit singulation, conveying, and orientation unit converts bulk fruit into a single-file stream with controlled spacing and orientation. Representative mechanisms include differential-speed belts, helical rollers, double-cone rollers, fruit cups or trays, brush rollers, and suspended-grip conveyors. Differential-speed belts provide high-speed separation; helical and double-cone rollers combine spacing with fruit rotation; cups facilitate weighing, identification, and precise discharge; and suspended conveying reduces contact between damage-sensitive fruit, such as strawberries and grapes, and the conveyor surface. Mechanism selection requires a balance among fruit-surface coverage, throughput, jamming risk, and impact damage [74,78,79] (Figure 3B) (Table 7).

Faheem et al. evaluated grape clusters weighing 0.48–0.53 kg at conveyor speeds of 0.4–1.0 m/s and accelerations of 6–12 m/s^2^. Changes in suspension force increased with speed and acceleration (R^2^ = 0.92), and collision with a rigid plastic box produced a maximum force change of 8.6 N, indicating that grape clusters susceptible to berry detachment require gradual acceleration and compliant receiving surfaces [78]. Pothula et al. employed a three-channel, multistage helical drive to singulate, rotate, and advance apples. At single-channel rates of 1 and 3 fruits/s, the complete fruit surface was imaged more than four times and at least 1.4 times, respectively, while the total throughput of the three-channel system exceeded 9 fruits/s. This configuration is suitable for online grading that requires full-surface defect inspection [13].

### 5.3. Online Inspection Unit

The online inspection unit comprises sensors, illumination or excitation sources, light-shielding structures, triggers, and acquisition interfaces. Sensor selection should be matched to the target attribute: machine vision is suitable for size, color, and surface defects; Vis/NIR spectroscopy for SSC, dry matter content, and maturity; hyperspectral imaging for joint spatial–spectral analysis; X-ray imaging for cavities, insect damage, and internal structural abnormalities; and weighing or mechanical sensors for auxiliary grading based on mass and firmness. Optical path length, tissue penetration depth, peel thickness, fruit orientation, surface moisture, temperature, illumination and conveyor vibration substantially affect online signals. Models should therefore be stress-tested across these presentation and environmental conditions rather than evaluated only under fixed laboratory settings. Adding more sensors is therefore not inherently beneficial; priority should instead be given to light shielding, positioning, synchronized triggering, routine cleaning and regular calibration [77,82,83,84,85,86,87,88] (Figure 3C) (Table 8).

Wang et al. developed an online near-infrared grading system for fresh long jujube that assigned three grades according to SSC. The overall grading accuracy for external validation samples was 86.7%, and the optimal model achieved an RPD of 2.8, indicating that compact spectral modules can support internal-quality classification [8]. Qiao et al. established an online visible–near-infrared system for strawberries transported by suspended grippers. A 1D-CNN-LSTM model predicted SSC with R^2^p = 0.963, RMSEP = 0.209 °Brix, and RPD = 5.332. This configuration reduced contact between damage-sensitive small fruit and the conveying surface, although stable gripping positions and a consistent transmission optical path remain essential [81].

### 5.4. Intelligent Classification and Control Unit

The intelligent classification and control unit converts sensor data into executable grading instructions according to a standard process which consists of acquisition, pre-processing or inference, identification of fruit type, determination of grade, adjustment of position and timing, and response of the controller. Industrial computers are suitable for multiview, multispectral, and deep-learning computations; controllers provide deterministic control of motors, cylinders, and electromagnetic actuators; and edge-AI devices are appropriate for space-constrained or low-power applications. The control system should associate each fruit ID with its encoder position and distance from the actuation station. End-to-end latency, missed-trigger rate, and stability during continuous operation should be reported alongside offline model accuracy [12,14,69,89,90,91,92] (Figure 3D) (Table 9).

Xu et al. deployed an improved YOLOv5 model in an automated apple-grading machine. The algorithm achieved a mean test-set accuracy of 90.6% and an inference speed of 59.63 frames/s, whereas the complete machine achieved a grading accuracy of 93% at a throughput of 4 fruits/s. These results indicate that model performance must be re-evaluated at the operating rate of the complete system [5]. Chen et al. combined a CNN detector with a SORT tracker to continuously associate multiple citrus targets without modifying the existing conveying line, achieving an overall online defect-detection accuracy of 93.66%. This detection–tracking–position-prediction architecture is suitable for production lines in which fruit moves continuously, and the actuation station is separated from the camera [14].

### 5.5. Sorting Actuators

Sorting actuators divert fruit according to the assigned grade and predicted arrival time. Common mechanisms include air jets, electromagnetic or pneumatic pushers, paddles, flaps or gates, cup discharge, multichannel lane diversion, and robotic compliant gripping. Air jets respond rapidly but are suitable only for small, lightweight fruit; paddles and flaps are appropriate for medium-mass fruit such as apples and citrus fruit; cup discharge can be readily coordinated with weighing and identification; and compliant robotic gripping is suitable for high-value, damage-sensitive fruit but incurs greater cost and longer cycle times. Contact mechanisms should incorporate curved surfaces, compliant materials, and cushioned drops to prevent secondary damage during actuation after the fruit has passed inspection [93,94,95,96,97,98] (Table 10). Because bruising can shorten shelf life and create sites for microbial infection, damage-free rate, delayed bruise incidence, decay incidence and marketable yield should be treated as co-primary performance indicators rather than secondary mechanical checks [2] (Figure 3E).

Zhang et al. developed a fresh-jujube grading machine with electromagnetically synchronized actuators. Image processing required 50 ms/frame, and actuator response required 40 ms. The machine diverted fruit into five size grades and one color grade, achieving an overall accuracy of 93.7%, a damage-free rate of 100%, an efficiency of 20 fruits/s, and a maximum productivity of 1400 kg/h. These results indicate the advantage of synchronizing high-speed electromagnetic actuation with conveyor timing [15]. Chen et al. designed a three-finger compliant gripper incorporating force feedback and slip detection. Activating force feedback reduced the apple-grasping damage rate from 20% to 0%. In controlled experiments, the compliant fingers with slip detection achieved an 80% grasping success rate without peel damage, indicating that compliant actuation is well suited to low-damage handling but still requires optimization between grasping reliability and fruit protection [80].

In an integrated grading line, the slowest or least reliable module constrains overall throughput. Even a highly accurate sensor offers limited commercial value if other operations, including cleaning, fruit presentation, calibration, actuation, sanitation, and maintenance, cause downtime or fruit damage. Complete-machine trials and life-cycle economic assessments are therefore essential for evaluating commercial viability.

## 6. Applications to Representative Fruit Categories

The following subsections group case studies by commodity and handling context, not by a single botanical classification. To support transferability, external validation must include variation in cultivar, season, origin, maturity, and storage history. Internal random splits are useful for model development but do not demonstrate generalizability under commercial conditions (Table 1).

### 6.1. Pome Fruits

Pome fruits, represented by apples and pears, must be graded according to both external conformity and consistent internal eating quality. Fruit diameter, shape, coloration ratio, and surface defects remain the basis of commercial grading, whereas soluble solids content (SSC), firmness, watercore, moldy core, and storage-related internal browning determine quality-based pricing and suitability for storage and transportation. The relatively large size of individual fruit facilitates continuous conveying in fruit cups or on double-cone rollers and enables rotational imaging. Nevertheless, interference from calyx and stem tissues, surface occlusion, asymmetric pear morphology, and spectral drift across cultivars and production seasons continue to reduce grading stability. Research on pome-fruit grading has therefore progressed from single size- or color-based decisions toward the coordinated application of multiview external inspection and spectroscopic internal-quality assessment [5,6,11,12].

**Figure 4:**
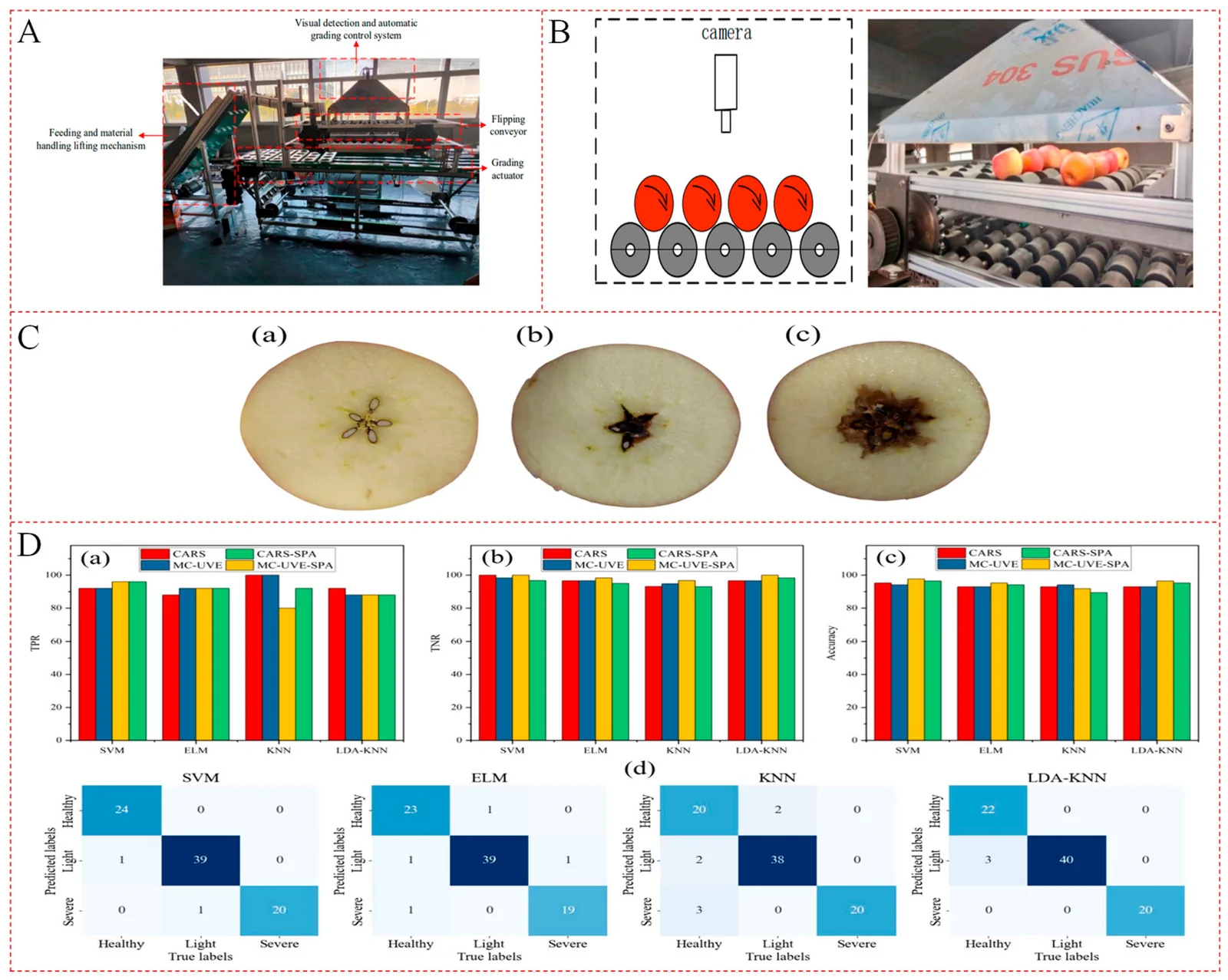
Representative applications in apple grading. (**A**) Four-lane apple-detection equipment [6]. (**B**) Roller turnover detection [6]. (**C**) Healthy (**a**), mildly affected (**b**), and severely affected (**c**) apples [16]. (**D**) Two-class classification results: TPR (**a**), TNR (**b**), accuracy (**c**), confusion matrix of prediction sets based on MC-UVE-SPA model (**d**) [16].

The engineering development of external apple grading is relatively advanced, and research has shifted from static single-fruit recognition toward parallel processing of multiple fruits, full-surface imaging, and closed-loop detection and actuation. Xu et al. incorporated an improved YOLOv5 model into a grading machine comprising elevator feeding, double-cone rollers for fruit rotation and conveying, and cup-based actuators, thereby classifying apples into three grades. The mean test-set grading accuracy was 90.6%, and the processing speed was 59.63 frames/s; in complete-machine experiments, the grading accuracy reached 93% at a throughput of 4 fruits/s [5]. Ji et al. further distinguished stems and calyx regions from true defects in multiview images of rolling apples and enhanced the multi-feature discrimination capability of YOLOv5s using a Swin Transformer. The model achieved an accuracy of 94.46% at 32 frames/s. In a complete-machine experiment involving 300 apples per grade and 900 apples in total, the mean grading accuracy was 94% at a throughput of 4 fruits/s. When the conveying speed was increased by 25%, motion blur reduced accuracy by 4.4 percentage points, indicating that production-line operation requires a balance among surface coverage, exposure time, and throughput [6] (Figure 4A,B). For applications in which defect area contributes to grade assignment, Liang et al. combined BiSeNet V2 semantic segmentation with a pruned YOLOv4 model. The mean pixel accuracy for defect segmentation reached 99.66%, while the online grading accuracy and F1-score for defective apples were 92.42% and 94.31%, respectively [12]. These results show that the main problem in external apple grading is not only to enhance the accuracy of single images but also to guarantee coverage of the fruit surface, identification tracking and consistent actuation time during continuous rotation.

Internal apple quality grading mainly focuses on sweetness, firmness and hidden defects and can use Vis/NIR reflectance or transmittance spectroscopy to give information not observed by machine vision. Fan et al. used a portable Vis/NIR device and a PLS model to predict apple SSC. The prediction-set R^2^, RMSEP, and RPD were 0.777, 0.561 °Brix, and 2.114, respectively. Independent laboratory validation yielded an R^2^ of 0.764 and an RMSEP of 0.672 °Brix, whereas recalibration using field data resulted in a field-prediction R^2^ of 0.690 and an RMSEP of 0.604 °Brix [11]. Guo et al. developed a portable transmittance system and constructed a CARS-CNN model that predicted SSC, firmness, pH, and watercore severity with correlation coefficients of 0.951, 0.824, 0.828, and 0.943, representing improvements of 3.0%, 11.2%, 11.2%, and 9.3%, respectively, over the corresponding full-spectrum models [99]. To detect externally invisible moldy core, Jiang et al. acquired dynamic online transmittance spectra from 340 ‘Red Fuji’ apples. A binary MC-UVE-SPA-LDA-KNN model achieved an area under the receiver operating characteristic curve (AUC) of 0.99 and an accuracy of 98.82%; three-class discrimination among healthy, mildly affected, and severely affected apples also yielded an AUC of 0.99 and an accuracy of 97.64% [16] (Figure 4C,D). Transmittance spectroscopy is therefore better suited to rejecting fruit with internal defects, whereas reflectance spectroscopy and portable devices facilitate sampled SSC measurements. Transfer to high-speed grading lines still requires control of model shifts caused by fruit diameter, temperature, and measurement position.

**Figure 5:**
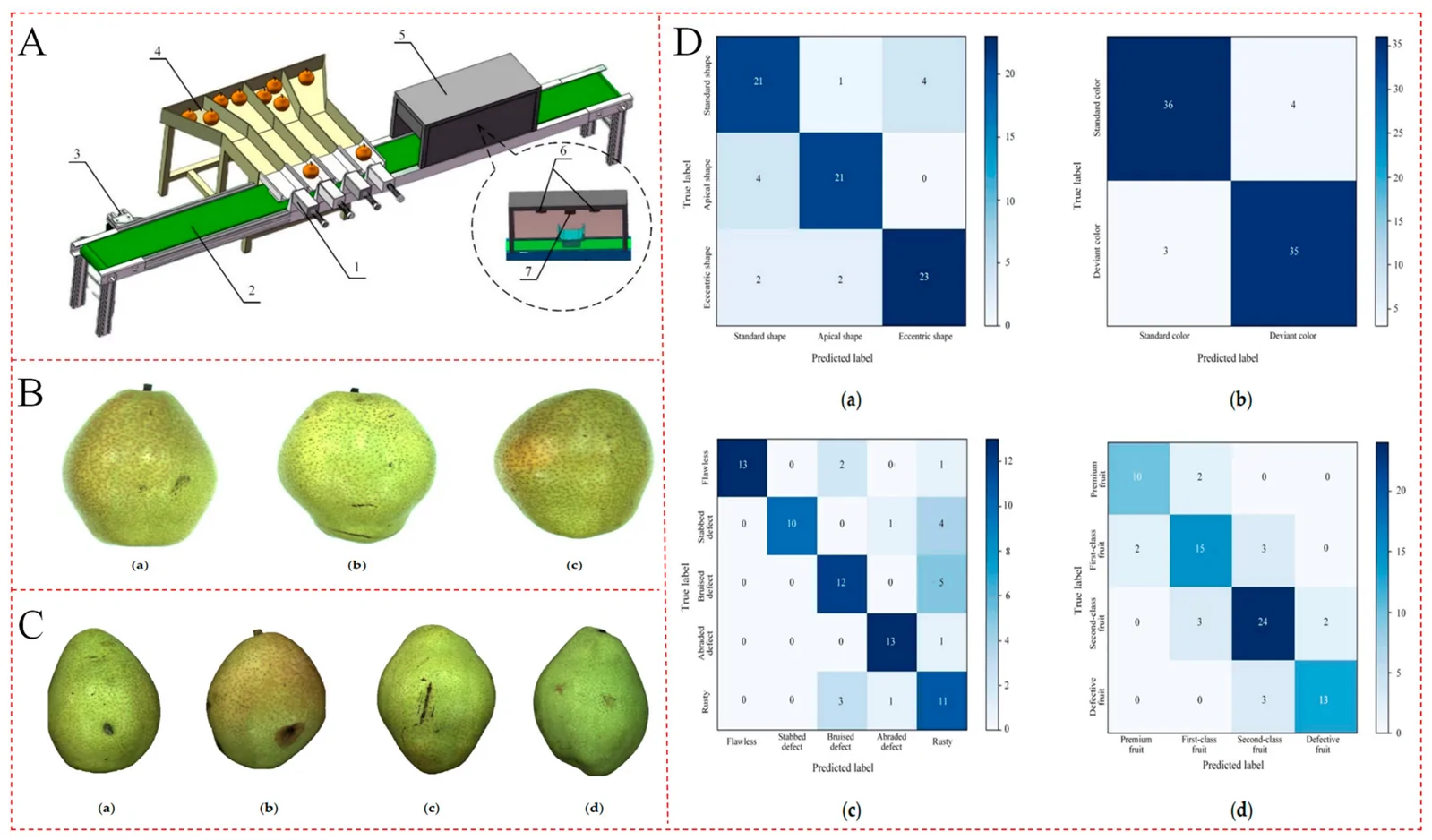
Representative applications in pear grading. (**A**) Pear appearance-inspection and grading system: 1, push–pull electromagnet; 2, conveyor; 3, motor; 4, storage box; 5, image-acquisition cabin; 6, light source; 7, CCD color camera [100]. (**B**) Standard (**a**), apical (**b**), and eccentric (**c**) pear shapes [100]. (**C**) Stabbed (**a**), bruised (**b**), abraded (**c**), and rusty (**d**) surface defects [100]. (**D**) Confusion matrices for shape (**a**), surface-color (**b**), surface-defect (**c**), and integrated (**d**) appearance-quality classifiers [100].

External pear grading differs from apple grading primarily because pears are asymmetric and exhibit conspicuous lenticels and russeting patterns. Consequently, single-view contours or color thresholds may misclassify natural surface textures as defects. Yang et al. decomposed pear appearance quality into three indices: fruit shape, surface color, and defects. Backpropagation neural networks or support vector machines were trained using symmetry and quasi-rectangularity descriptors, R/G-channel statistics, and gray-level co-occurrence matrix features extracted from defect regions. Classification accuracies for shape, color, and defects were 83.3%, 91.0%, and 76.6%, respectively, while rule-based integrated grading achieved an accuracy of 80.5% [100] (Figure 5A–D). The study also incorporated conveying, position sensing, image inspection, and push–pull solenoid sorting units but could not acquire images of the complete pear surface. These results indicate that multi-index grading rules can improve the interpretability of grade assignments but are susceptible to the accumulation of errors across cascaded classifiers. This approach is suitable for small- and medium-scale grading lines handling a single cultivar under stable standards, whereas high-throughput applications require multiview image fusion and end-to-end joint classification.

Internal pear-quality assessment must also address inconsistent spectral sampling positions caused by asymmetric fruit morphology, together with differences in peel color and stone-cell content among cultivars. Xu and Mishra employed deep transfer learning to locate a symmetric region on the ventral surface of pears, extracted spectra near its centroid, and predicted dry matter content using PLS. The ventral region was detected in all 111 hyperspectral images, with an intersection over union (IoU) of 0.82, and an 11 × 11-pixel sampling window yielded a dry matter RMSEP of 0.77% [101]. Zhang et al. acquired 398–1004 nm hyperspectral data from 1200 samples representing six pear cultivars, applied first-derivative and standard normal variate (FD-SNV) preprocessing, selected characteristic wavelengths using CARS, and developed LS-SVM models. The SSC model for ‘Huangguan’ pears achieved an RPD of 5.4, the multicultivar firmness model achieved an RPD of 3.4, and BPNN classification accuracies for all six cultivars exceeded 99% in both the calibration and prediction sets [102]. In an earlier study, Goke et al. used a commercial NIR instrument to predict dry matter content and SSC in ‘d’Anjou’ and ‘Bartlett’ pears. Cultivar-specific calibration R^2^ values were 0.940 and 0.860 for dry matter content and 0.908 and 0.839 for SSC, respectively; under independent validation, R^2^ values ranged from 0.722 to 0.901 for dry matter content and from 0.651 to 0.844 for SSC [103]. Collectively, these studies indicate that internal-quality grading of pears depends not only on the algorithm but also on the sampling region, cultivar coverage, and the inclusion of maturity and storage-related variation in the calibration set.

For commercial deployment, apples favor rotational RGB inspection with a targeted transmittance channel for internal-defect rejection, whereas pears require morphology-aware view selection before external and internal measurements are fused. Claims of transferability should rely on independent cultivar, season, site and line tests and should report throughput, missed detections, fruit damage and long-term stability.

### 6.2. Stone Fruits

Stone fruits, represented by peaches, plums, and apricots, ripen rapidly, soften markedly, and develop bruises that readily propagate from the skin into subsurface tissues. Fruit diameter, ground color, and coloration can be measured rapidly by machine vision, but external ripeness does not necessarily correspond to appropriate levels of soluble solids content (SSC), dry matter content, acidity, and firmness. Sutures, pubescence, surface bloom, and natural spots also interfere with defect recognition. Moreover, newly formed minor bruises are often visually imperceptible and may subsequently develop into browning and decay as the fruit continues to soften after grading. Applications to stone fruits should therefore integrate external appearance and maturity grading, prediction of internal eating quality, rejection of early mechanical injury, and low-contact conveying, with target attributes selected according to the skin characteristics and intended markets of peaches, plums, and apricots [17,18,104,105,106,107].

Internal-quality grading of peaches must first determine whether the fruit is suitable for harvest, transportation, or immediate consumption. Portable Vis/NIR instruments are appropriate for rapid pregrading at harvest and at the entrance to packing lines. Minas et al. expanded the quality range of the calibration samples through a crop-load × fruit-development-stage experiment and used a single 729–935 nm handheld Vis/NIR scan to predict dry matter content, SSC, and the index of absorbance difference. The prediction models achieved R^2^ values of 0.98, 0.96, and 0.96 and RMSEP values of 0.41%, 0.58%, and 0.08, respectively [44]. Field validation further showed that a higher crop load reduced dry matter content and SSC and delayed maturity, indicating that spectral grading can support both harvest-batch allocation and sorting according to flavor potential and transportation distance. Masuda et al. trained a CNN using 1521 RGB images of ‘Hakuho’ peaches to simultaneously diagnose seven traits: skin color, firmness, sugar content, colorless early softening, split pit, watercore, and peach fruit moth injury. Correlation coefficients were −0.55 between skin color and colorless early softening and 0.38 between watercore and sugar content, and explainable artificial intelligence was used to localize the image regions contributing to model decisions [104]. Online deployment nevertheless requires cultivar, canopy position, production season, and fruit temperature to be represented in the calibration set; a high goodness of fit under conditions from a single orchard should not be interpreted as general grading capability.

**Figure 6:**
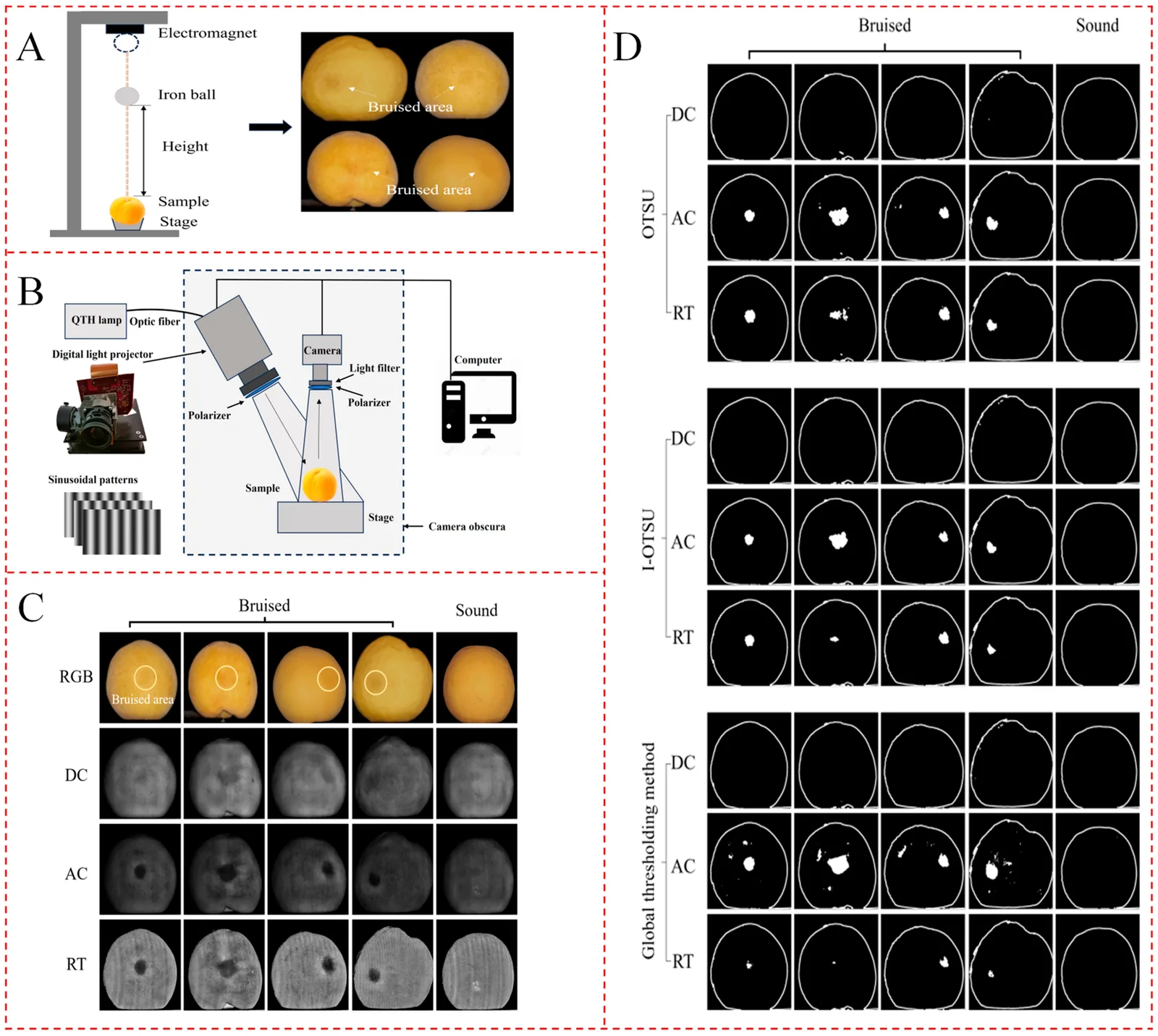
Representative applications in peach grading. (**A**) Impact-test device and slightly bruised yellow peaches [17]. (**B**) Multispectral structured-illumination reflectance imaging system [17]. (**C**) RGB, direct-component, alternating-component and ratio images of five representative fruits; white circles indicate bruises [17]. (**D**) Segmentation results from Otsu, improved Otsu and global-thresholding algorithms [17].

Another important application in peaches is the rejection of early minor bruises caused during sorting, handling, and transportation. For this task, sensing depth and robustness to nonuniform illumination are more important than conventional RGB surface recognition. Wu et al. conducted controlled impact experiments on 300 yellow peaches and acquired multispectral structured-illumination reflectance images at 700 nm and a spatial frequency of 0.10 cycles/mm. Ratio images derived from the alternating-current and direct-current components enhanced subsurface bruises, and an improved Otsu algorithm achieved an overall detection accuracy of 96% across all samples [17] (Figure 6A–D). The method highlighted bruised regions that were not yet visually apparent, although lenticels, stems, and sutures still caused segmentation errors, and the current device relied primarily on static acquisition. Structured-light imaging is therefore appropriate for secondary inspection of latent injury in high-value peaches. Integration into a continuous grading line will require reduced-phase demodulation, synchronized high-speed projection, and low-vibration fruit supports to shorten the acquisition time per fruit.

Plum maturity grading commonly relies on changes in peel color from green to yellow-red or purple-red. However, overall coloration, surface bloom, and cultivar differences reduce the stability of a single color threshold. Kaur et al. acquired 140 images of ‘Satluj Purple’ plums and assigned the samples to four stages: mature green, color break, fully colored, and overripe. For each stage, 25 images were used for calibration and 10 for validation. Errors between the image-derived and manually measured major and minor axes were below 2.4%. Fruit acidity showed an R^2^ of 0.9966 with mean green-channel intensity, while SSC showed an R^2^ of 0.8464 with the R/G ratio [105]. However, 20% of the validation samples were misclassified when texture features alone were used, indicating that color is suitable for low-cost preliminary maturity screening, whereas texture should remain supplementary rather than serve as the sole grading variable.

**Figure 7:**
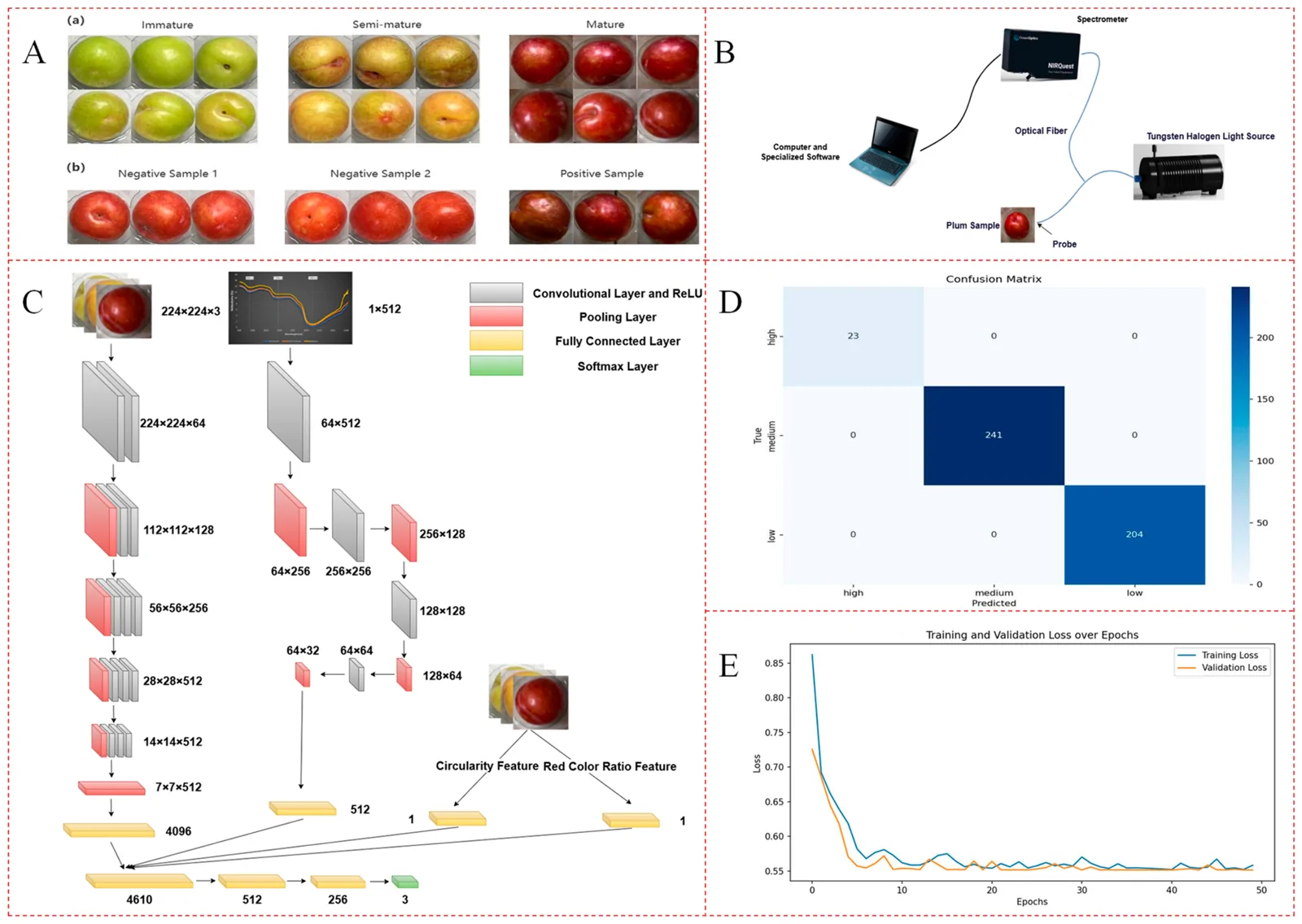
Representative applications in plum grading. (**A**) Positive (**a**) and negative (**b**) samples at three maturity levels [106]. (**B**) Near-infrared spectral system [106]. (**C**) Multimodal fusion grading model diagram [106]. (**D**) Confusion matrix for the multimodal fusion model [106]. (**E**) Training-loss curve [106]. In (**C**), numbers give input dimensions and layer sizes; the final three outputs correspond to the three quality grades.

When external plum coloration and internal ripeness are asynchronous, fusion of RGB images and visible–near-infrared spectra provides a more comprehensive basis for commercial grade determination. Liu et al. investigated 780 samples of ‘March’ and ‘Sanhua’ plums, extracting RGB appearance features with VGG16 and processing 350–1700 nm spectra with a one-dimensional CNN before fusing the two representations through a fully connected network. On the test set, the image and spectral models achieved accuracies of 85.71% and 83.33%, respectively, whereas the fusion model achieved 100% accuracy, precision, and recall [106] (Figure 7A–D). In an adversarial experiment in which 33% of the training labels were randomly flipped, the fusion model retained a test accuracy of 79.06%, indicating a degree of robustness to label noise. Nevertheless, the 100% result was obtained from an internal split of a single dataset and requires verification across orchards, production seasons, storage stages, and independent production lines. Engineering implementation must also ensure same-fruit registration between images and spectra and synchronize the operating rates of the two sensors.

**Figure 8:**
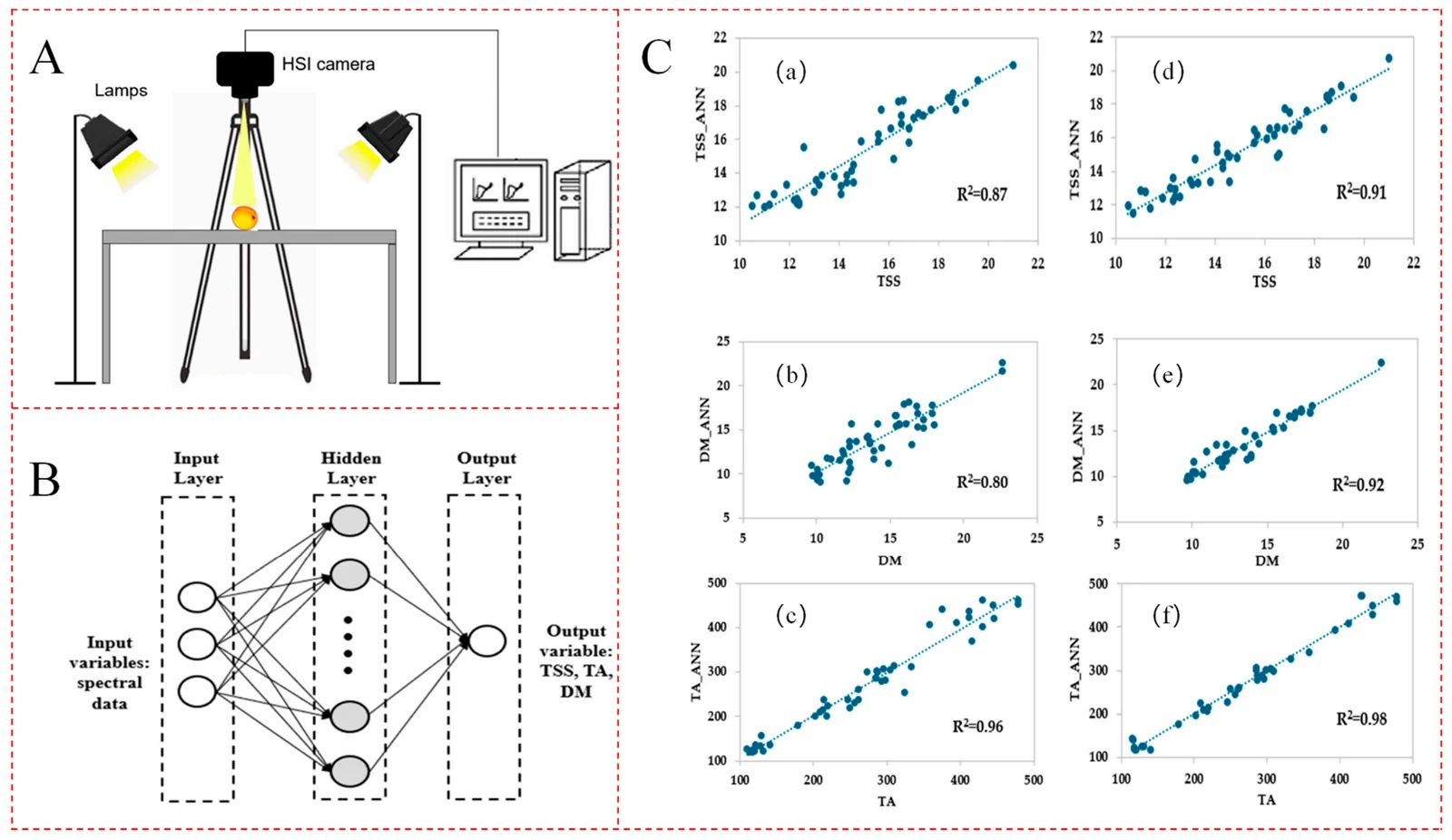
Representative applications in apricot grading. (**A**) Vis/NIR hyperspectral imaging system [18]. (**B**) ANN-MLP architecture [18]. (**C**) Predicted vs. experimental values of total soluble solid content (TSS), titratable acidity (TA), and dry matter (DM) using external Vis/NIR spectrophotometer dataset (**a**–**c**), external HSI dataset (**d**–**f**) and the optimal ANN topologies. The coefficients of determination (R^2^) are reported [18].

Apricots are graded for fresh consumption and for processing into dried products, preserves, and juice; consequently, soluble solids content (SSC), dry matter content, and titratable acidity determine product allocation more directly than fruit diameter alone. Amoriello et al. developed multicultivar, cross-year models using samples from 17 apricot cultivars and two production seasons. The dataset comprised 204 fruits and 408 Vis/NIR spectra acquired from opposite sides, while four additional cultivars were used for external validation. A portable spectrometer combined with an ANN-MLP model predicted SSC, dry matter content, and titratable acidity with test-set R^2^ values of 0.855, 0.857, and 0.681, respectively; the corresponding 400–1000 nm hyperspectral imaging models improved these values to 0.904, 0.918, and 0.811 [18] (Figure 8A–C). Liu et al. further combined spectra acquired from opposing views with ensemble learning to predict the internal quality of peaches, apricots, and cherries. For apricots, the MLP-SG-XGBoost model achieved an R^2^ of 0.9251 for SSC, while the LGR-SG-LightGBM model achieved an R^2^ of 0.9206 for titratable acidity [107]. These findings indicate that internal-quality grading of apricots is well suited to multi-attribute spectroscopic assessment. Portable instruments are less expensive and appropriate for sampling-based inspection and raw-material reception, whereas hyperspectral imaging provides more comprehensive information for refined allocation of high-value fruit but requires characteristic-wavelength selection to reduce the cost and data-processing burden of online systems.

Commercial stone-fruit systems should combine maturity-based allocation with latent-bruising rejection on compliant, low-drop conveyors. Evidence should be judged by external validation, inspection time, throughput and post-grading damage rather than only by R^2^ or accuracy.

### 6.3. Citrus Fruits

Citrus fruits include sweet oranges, mandarins, pomelos, and grapefruits, although research on intelligent postharvest grading has focused primarily on oranges and mandarins. Oranges are relatively large and have tightly adhering peels, making them suitable for continuous rotation on roller conveyors and multiview external inspection. Mandarins have loose, easy-peeling rinds and require assessment not only of color and surface defects but also of soluble solids content (SSC), peel puffing, and fruit-fly infestation. Although the thick citrus peel improves resistance to transportation damage, it attenuates visible–near-infrared penetration into the flesh, potentially creating discordance between external coloration and internal flavor or between visually normal surfaces and incipient decay. Representative applications have therefore progressed from diameter- and color-based sorting toward a hierarchical strategy comprising online screening for visible defects, spectroscopic verification of internal quality, and rejection of latent diseases and insect damage [14,19,108,109,110,111,112,113].

High-speed commercial grading of oranges first requires stable detection of mechanical injury and disease lesions under fruit rolling, mutual occlusion, and conveyor vibration. RGB vision offers low cost, high speed, and straightforward integration into existing production lines. Chen et al. acquired 40 videos at 30 Hz from 300 oranges and constructed a dataset of 2400 images. A Mobile-Citrus detector combining MobileNet-V2 and PANet was paired with a SORT tracker to aggregate classifications of the same fruit across consecutive frames, enabling online discrimination among sound fruit, mechanically damaged fruit, and fruit with peel lesions. The overall detection-and-tracking accuracy reached 93.66% [14]. With 8–20 fruit per frame, the mean processing time was 23 ms, equivalent to 43 frames/s. Misclassification rates for sound and defective fruit were 2.53% and 3.76%, respectively, while the corresponding missed-detection rates were 3.68% and 2.83%. The method is suitable for high-speed preliminary screening of visible defects in packing houses. However, robotic rejection remained at the conceptual design stage, and fruit near the conveyor edges might not present their complete surfaces. Practical systems therefore require multiple cameras or controlled fruit-rotation mechanisms to eliminate blind areas.

**Figure 9:**
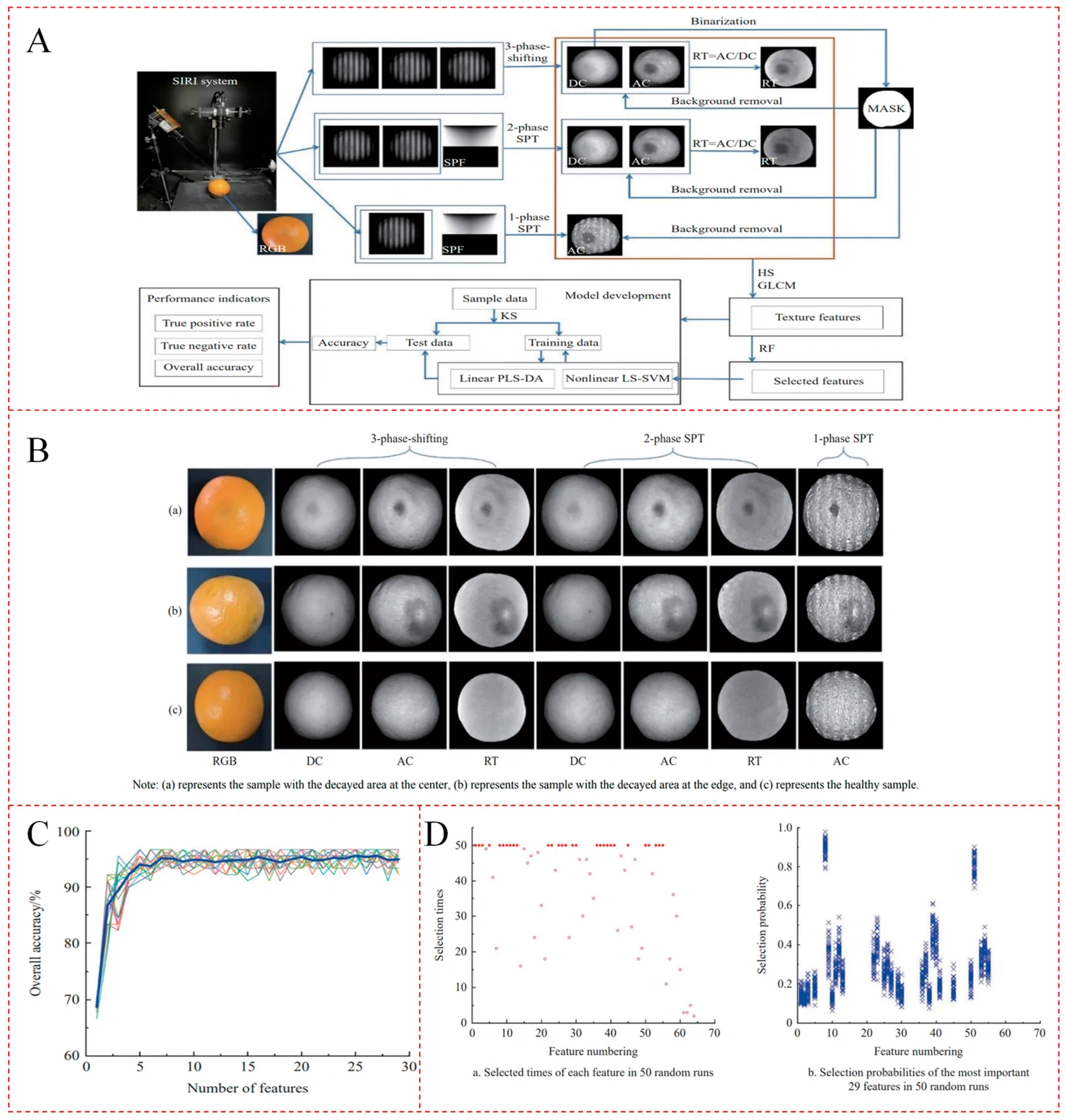
Representative applications in orange grading. (**A**) Workflow for detecting decayed oranges [109]. (**B**) RGB, direct-component, alternating-component and ratio images obtained using three demodulation methods [109]. (**C**) Classification accuracy as a function of the number of selected features [109]. (**D**) Random-frog feature-selection results showing selection counts across 50 runs (left, red circles) and selection probabilities for the 29 most frequently selected features (right, blue crosses) [109].

When peel coloration resembles incipient green mold, citrus canker, or mild decay, conventional RGB imaging provides insufficient contrast, making hyperspectral and structured-light imaging more suitable for secondary inspection of latent defects. Li et al. acquired 975.18–2196.2 nm hyperspectral images from 30 sound navel oranges, 30 fruit with canker, and 30 fruit infected with green mold. An LSTM model distinguished sound peel, canker lesions, green-mold spores, and hyphal regions. The full-spectrum model using all 217 bands achieved a test accuracy of 92.81%, with a testing time of 46.21 s per fruit. Selecting 21 wavelengths using independent component analysis (ICA) increased accuracy to 95.01% and reduced the testing time to 3.71 s. Further compression to six wavelengths using ICA-GA retained an accuracy of 93.41% while reducing the testing time to 1.26 s [108]. Cai et al. acquired three phase-shifted structured-light images at a spatial frequency of 0.25 cycles/mm and recovered direct-component (DC), alternating-component (AC), and ratio (RT) images using a two-phase spiral phase transform. An LS-SVM model constructed from seven texture features extracted from the RT images achieved a mean classification accuracy of 95.1% for oranges with incipient decay [109] (Figure 9A–D). Collectively, these studies indicate that informative-wavelength selection and rapid demodulation are critical for converting high-dimensional laboratory imaging into online secondary-inspection units. Nevertheless, 1.26 s per fruit remains too slow for high-throughput main lines. A more practical strategy is to use hyperspectral imaging to identify a small number of sensitive wavelengths and subsequently develop multispectral or reduced-phase structured-light devices.

Mandarin cultivars vary substantially in peel color, oil-gland characteristics, and surface brightness. External-defect detection must therefore accommodate locally low-contrast regions, while eating-quality grading should avoid equating good coloration directly with high sugar content. Li P. et al. proposed a sliding-window segmentation method that required no brightness correction for 100 real-world images of ‘Wokan’ mandarins. With a 100 × 100-pixel window, surface-defect detection required 85.3 ms per fruit and achieved a recognition rate of 97.5%. Reducing the window size to 20 × 20 pixels increased the recognition rate to 98.9% but also increased the computational demand [111]. For coordinated grading of external and internal quality, Zhang et al. acquired 380–1030 nm hyperspectral data from 550 ‘Nanfeng’ mandarins representing five categories: anthracnose, black spot, decay, scarring, and sound fruit. A CARS-CNN model achieved an overall defect-classification accuracy of 97.27%, while an SSC-CNN model developed using 150 sound fruits yielded R^2^ = 0.9290, RMSEP = 0.3772 °Brix, and RPD = 3.7655 [110]. Kim et al. further acquired 648 sets of 400–1000 nm hyperspectral data from the stem and calyx ends of 324 Satsuma mandarins. After outlier removal, CARS-PLSR selected 51 effective wavelengths for SSC prediction and achieved R^2^ = 0.75 and RMSEP = 0.559 °Brix on an independent prediction set [113]. These findings support the use of RGB imaging for low-cost preliminary defect screening followed by sugar-content estimation at selected wavelengths. However, the substantial variation in model performance among studies indicates that cross-cultivar calibration and compensation for peel thickness remain major constraints on internal-quality grading.

**Figure 10:**
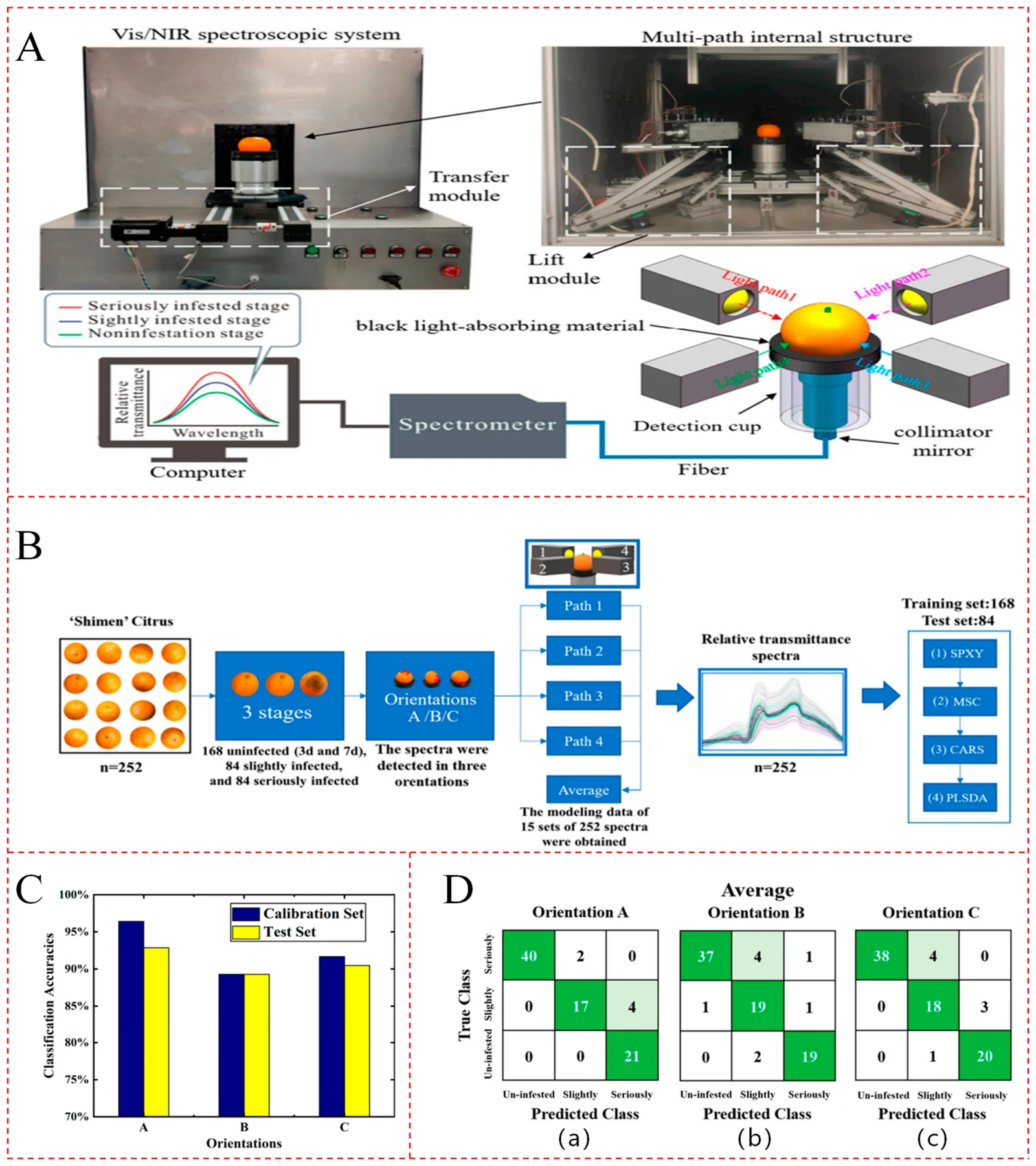
Representative applications in citrus grading. (**A**) Vis/NIR spectral-acquisition system [19]. (**B**) Data-collection and model-development workflow [19]. (**C**) Training- and test-set accuracies for four-path average spectra under three fruit orientations [19]. (**D**) PLS-DA test set results for average spectra of four paths in three fruit orientations. (a) Orientation A, (b) Orientation B, and (c) Orientation C [19]. In (**A**), light-path numbers 1–4 identify the four illumination paths. The red, blue and green schematic spectra indicate seriously infested, slightly infested and uninfested fruit, respectively. In (**B**), numbers 1–4 in the inset also identify the four light paths. In (**C**), dark-blue and yellow bars represent the calibration and test sets, respectively. In (**D**), each matrix entry gives the sample count for the corresponding true and predicted classes.

Mandarins are also affected by peel puffing and fruit-fly infestation, which can produce externally normal but internally abnormal fruit. If grading is based solely on color and diameter, affected fruit may enter fresh-market lots and deteriorate rapidly during distribution. Sun et al. compared healthy and peel-puffed ‘Iwasaki’ Satsuma mandarins harvested at three dates and characterized their peel structure using X-ray CT and laser-scattering imaging. Peel puffing reduced the thicknesses of both the flavedo and albedo and increased albedo porosity. The reduced scattering coefficient of intact peel-puffed fruit was lower than that of healthy fruit, and this optical difference was validated using an independent ‘Goku Wase’ cultivar, enabling separation of healthy and peel-puffed fruit at all selected wavelengths [112]. Li D. et al. investigated 252 ‘Shimen’ citrus fruits, including 126 artificially infested with fruit flies, using a 350–1100 nm transmittance system comprising four optical paths separated by 90°. Spectra averaged across the four paths yielded overall accuracies of 92.9%, 89.3%, and 90.5% for fruit positioned stem-up, horizontally, and stem-down, respectively. The optimal model achieved a kappa coefficient of 0.89 and accuracies of 95.2%, 80.1%, and 100.0% for uninfested, mildly infested, and severely infested fruit, respectively [19] (Figure 10A–D). The markedly lower accuracy for mild infestation indicates that the principal challenge in grading internal diseases and insect damage is not recognizing fruit that has already decayed but obtaining sufficiently stable transmission signals before symptoms develop. Multiple optical paths reduce variability associated with the random location of infestation but increase the complexity of the optical structure, same-fruit registration, and throughput control.

Citrus systems are best organized as fast RGB screening followed by selected-band or multipath inspection of ambiguous or internal defects. Commercial evidence should include mild-defect recall, full-surface coverage, throughput, cross-cultivar validation and long-term calibration drift.

### 6.4. Kiwifruit and Mango

For climacteric fruits, commercial grade is not a static label fixed at harvest but is jointly determined by current maturity, postharvest ripening rate, and the target marketing window. Kiwifruit and mangoes are representative examples. Kiwifruit softens rapidly after harvest and has a narrow firmness window for consumption; fruit from the same batch may therefore diverge substantially in firmness and sugar content during storage. Mango peel color is strongly cultivar-dependent, and green fruit may already be physiologically mature, while maturity at harvest determines sweetness, aroma, and shelf life after ripening. Grading of climacteric fruit should consequently progress from external conformity alone toward a sequential framework comprising harvest-maturity identification, storage-stage classification, and allocation according to the eating-ripe window and remaining shelf life. Inspection results should also be linked to ripening treatments, cold storage, transportation distance, and intended sales timing [114,115,116,117,118,119].

**Figure 11:**
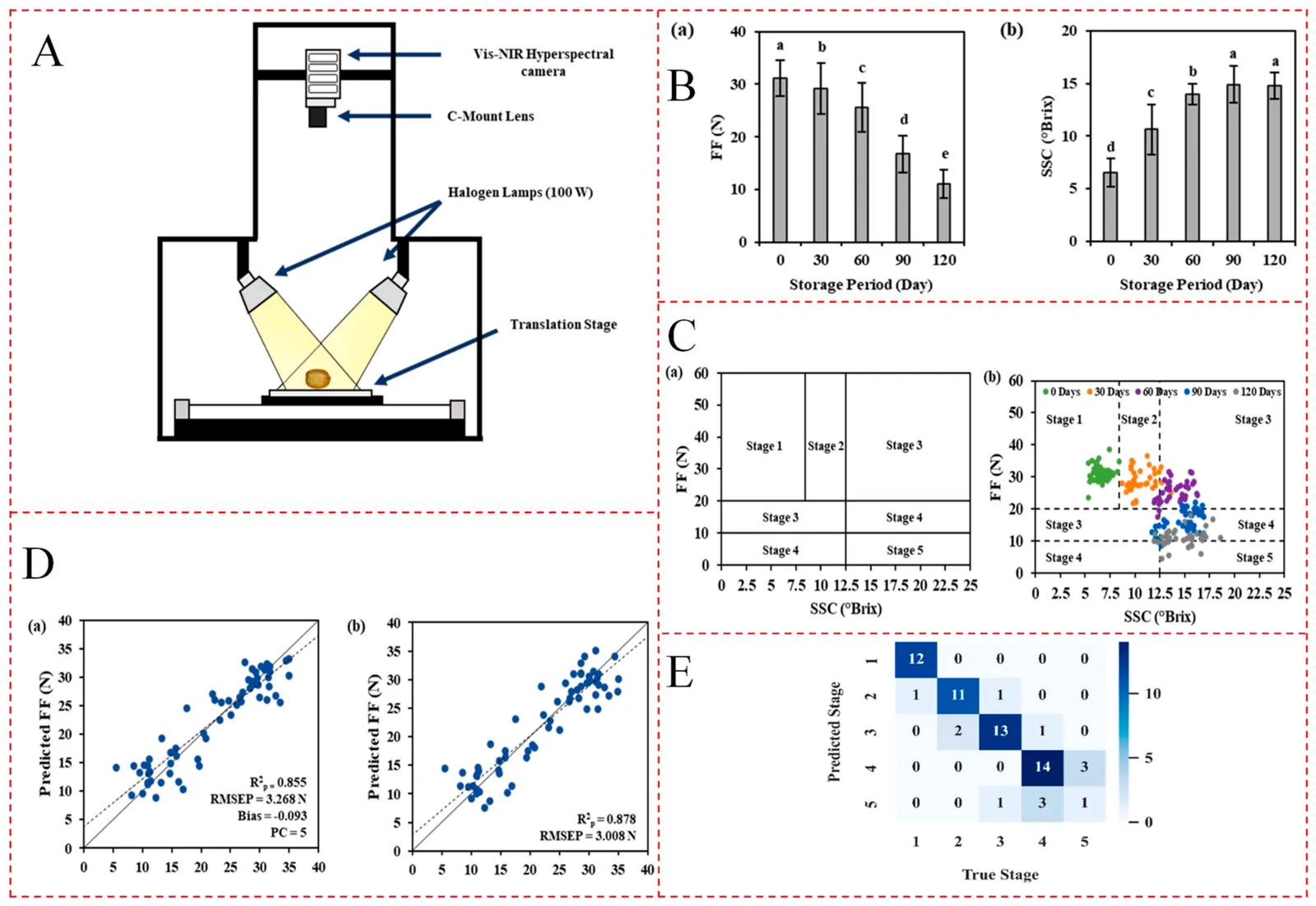
Representative applications in kiwifruit grading. (**A**) Vis/NIR hyperspectral imaging system [114]. (**B**) Duncan’s post hoc comparison results of quality attributes in kiwifruits according to the storage period: (a) FF, and (b) SSC [114]. Within each panel, different lowercase letters above the bars indicate significant differences among storage periods according to Duncan’s post hoc test. (**C**) Ripeness criteria and distribution classified according to the FF and SSC: (a) Definition of ripeness stage criteria; (b) Sample distribution by storage period [114]. (**D**) Validation results of optimal models to predict the firmness of kiwifruits: (a) PLSR model with 2n-order derivative preprocessing and (b) SVMR model with 2nd-order derivative preprocessing [114]. Dots represent individual samples. Solid lines indicate the 1:1 reference lines (predicted = measured), whereas dashed lines show the fitted regression lines. (**E**) Confusion matrix for SVMR ripeness classification [114].

Kiwifruit grading must simultaneously characterize flesh firmness, soluble solids content (SSC), and storage stage because external color alone cannot reliably distinguish firm, ready-to-eat, and overripe fruit. Lee et al. defined five postharvest ripening stages for kiwifruit stored at low temperature for 0–120 d and developed regression and classification models using visible–near-infrared hyperspectral imaging. An SVMR model with second-derivative preprocessing predicted flesh firmness with R^2^p = 0.878, RMSEP = 3.008 N, and RPD = 2.721, while an MSC-SVMR model predicted SSC with R^2^p = 0.940, RMSEP = 0.898 °Brix, and RPD = 4.055. The highest stage-classification accuracies achieved by PLS-DA and SVMC were 91.463% and 91.548%, respectively [114] (Figure 11A–E). These results indicate that hyperspectral imaging can jointly map firmness and SSC to shipping stages, although adjacent ripening grades remain difficult to distinguish. Engineering deployment should prioritize the selection of a small number of sensitive wavelengths and verification of measurement repeatability under high-speed conveying conditions.

For ready-to-eat supply chains, kiwifruit grading must extend beyond measuring current firmness to predicting the remaining eating-ripe period. Ding et al. collected Fourier-transform near-infrared spectra from 413 kiwifruit within the critical firmness range of 10–40 N. Following first-derivative preprocessing, competitive adaptive reweighted sampling, and support vector regression optimization, the FD-CARS-SVR radial-basis-function model achieved a prediction-set R^2^ of 0.92, an RMSEP of 0.40, and an RPD of 3.48, substantially outperforming the full-spectrum radial-basis-function model [116]. Zhang et al. monitored mass loss, firmness, SSC, ascorbic acid, titratable acidity, color difference, and sensory scores at 0, 5, 10, 15, and 20 °C. Zero-order reaction models achieved R^2^ values of 0.892–0.985, Ball temperature models achieved R^2^ values of 0.947–0.991, and the combined models limited prediction errors for quality attributes to within 6.5% [115]. The former approach supports online identification of ready-to-eat firmness, whereas the latter estimates remaining shelf life as a function of storage temperature. Their combination could enable kiwifruit to be allocated to long-distance transportation, short-term ripening, or immediate ready-to-eat sale.

The principal challenge in grading mangoes by harvest maturity is that peel color and internal dry matter accumulation are asynchronous. This problem is particularly pronounced in cultivars that remain green at maturity, for which color- or firmness-based sorting may result in premature harvesting. Shah et al. collected 240 hard-green mangoes over two consecutive harvest seasons, encompassing two cultivars and three harvest stages, and used a handheld 400–1100 nm near-infrared microspectrometer to directly classify the fruit as mature or immature. An indirect approach that first predicted dry matter content and then applied a threshold achieved a maximum accuracy of 55.9%, whereas direct KNN classification reached 88.2% [117]. On-site grading should therefore directly learn the decision boundary between fruit that can and cannot ripen normally. However, dry matter thresholds depend on cultivar and production region, necessitating external validation across seasons and orchards.

**Figure 12:**
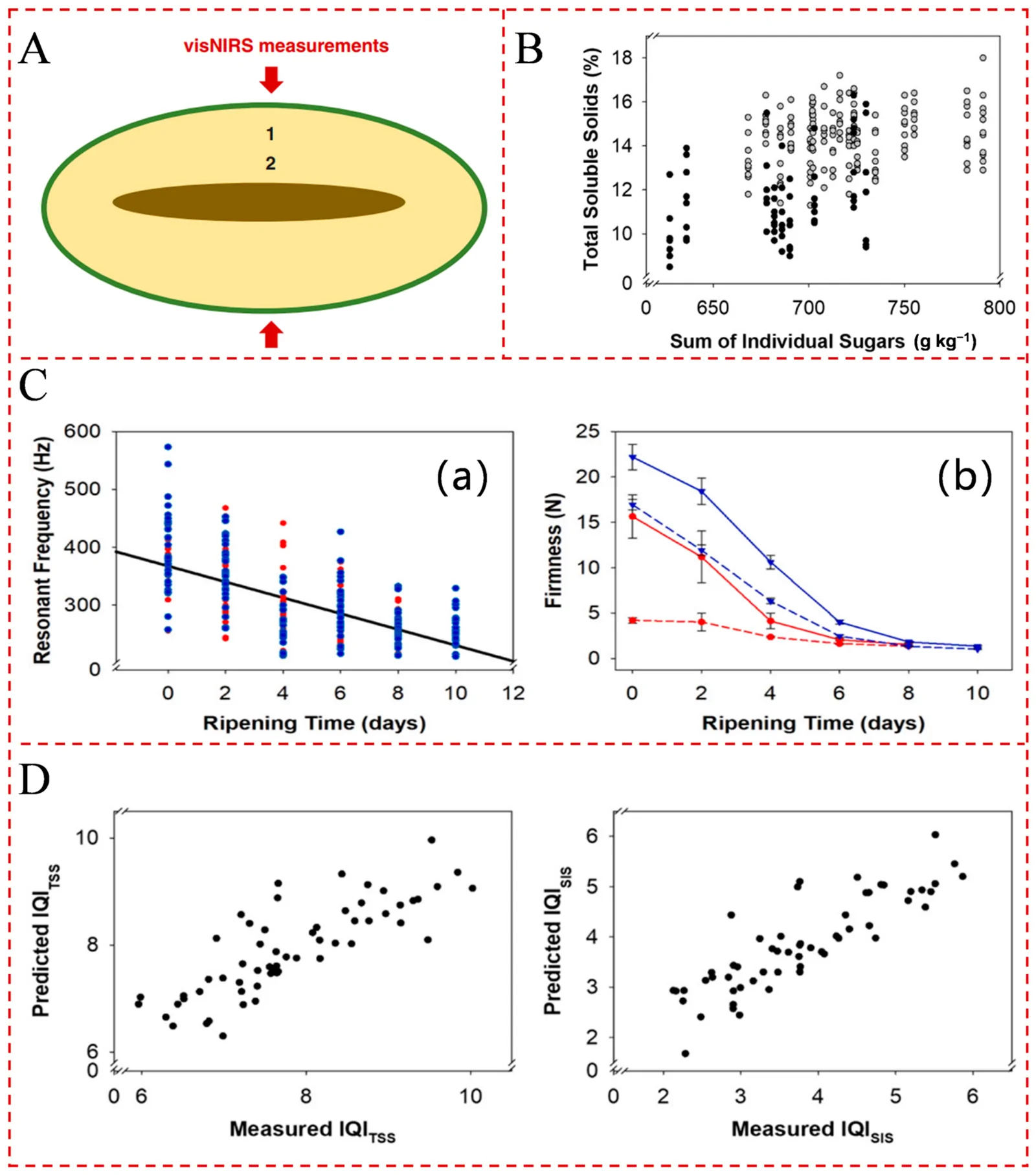
Representative applications in mango grading. (**A**) Measurement locations. Labels 1 and 2 identify outer and inner pulp, respectively; green outlines the skin, and brown denotes the stone. Red arrows mark the Vis/NIR measurement sites [118]. (**B**) Total soluble solids versus the sum of individual sugars in mangoes from Peru (gray) and Brazil (black) [118]. (**C**) Ripening-related changes in (a) resonant frequency and (b) destructively measured firmness. Blue and red represent ‘Kent’ and ‘Keitt’, respectively. In (a), dots represent measurements and the black line shows a linear fit. In (b), solid and dashed lines correspond to outer- and inner-pulp firmness, respectively [118]. (**D**) Predicted versus measured internal-quality index for Vis/NIR spectroscopy models developed using ‘Kent’ mangoes from Peru and the Dominican Republic [118].

During ripening and distribution, the grading target for mangoes shifts from harvest maturity alone to integrated quality determined by firmness, sugar content, flesh color, and volatile compounds. O’Brien et al. compared visible–near-infrared spectroscopy (Vis/NIR spectroscopy) with laser Doppler vibrometry for ‘Kent’ and ‘Keitt’ mangoes. A Vis/NIR spectroscopy-PLSR model predicted the internal quality index of ‘Kent’ mangoes with R^2^p = 0.729 and RMSEP = 0.532, whereas laser Doppler vibrometry yielded R^2^ < 0.5 for firmness prediction in both cultivars. These findings indicate that a linear decrease in resonance frequency does not adequately represent the exponential softening of mango flesh [118] (Figure 12A–D). Huang et al. fused machine vision with a colorimetric sensor array to classify mangoes stored at 12 ± 0.5 °C and 85–90% relative humidity into three stages: mature green, fully ripe, and overripe. The fused SVC model achieved training- and prediction-set accuracies of 98.75% and 97.5%, respectively. For the fused SVR model, the training/prediction correlation coefficients were 0.9051/0.8897 for firmness and 0.9515/0.9241 for soluble solids content [119]. Multimodal fusion is suitable for storage-room monitoring or prepacking verification, but colorimetric sensor arrays require reaction time and consumables. High-speed main lines are therefore better served by Vis/NIR spectroscopy as the primary modality, with visual and olfactory information reserved for ambiguous samples.

Kiwifruit and mango grading should link maturity and quality measurements to a stated logistics window while recording cultivar, batch, temperature and inspection time. General claims require independent season and site validation and demonstrated gains in shelf-life allocation or loss reduction.

### 6.5. Sweet Cherries, Blueberries, and Table Grapes

Small fruits generally share several characteristics, including small individual size, high batch volumes, thin skins, and high sensitivity to impact. However, the grading unit and priority attributes differ among sweet cherries, blueberries, and table grapes. Sweet cherries are typically evaluated individually for cracking, decay, deformity, and fruit diameter. Blueberries require small-target maturity classification on bushes or conveyor lines, together with the rejection of early bruising. Table grapes are commonly graded as whole clusters according to maturity, sugar–acid levels, cluster integrity, and berry condition. Previous research has demonstrated that visible–near-infrared spectroscopy combined with machine learning can support both maturity-stage discrimination and soluble solids content assessment [120]. Small-fruit grading is therefore better implemented through an integrated strategy comprising high-speed visual screening of external quality, spectroscopic verification of internal quality or latent injury, and compliant conveying with low-drop diversion.

**Figure 13:**
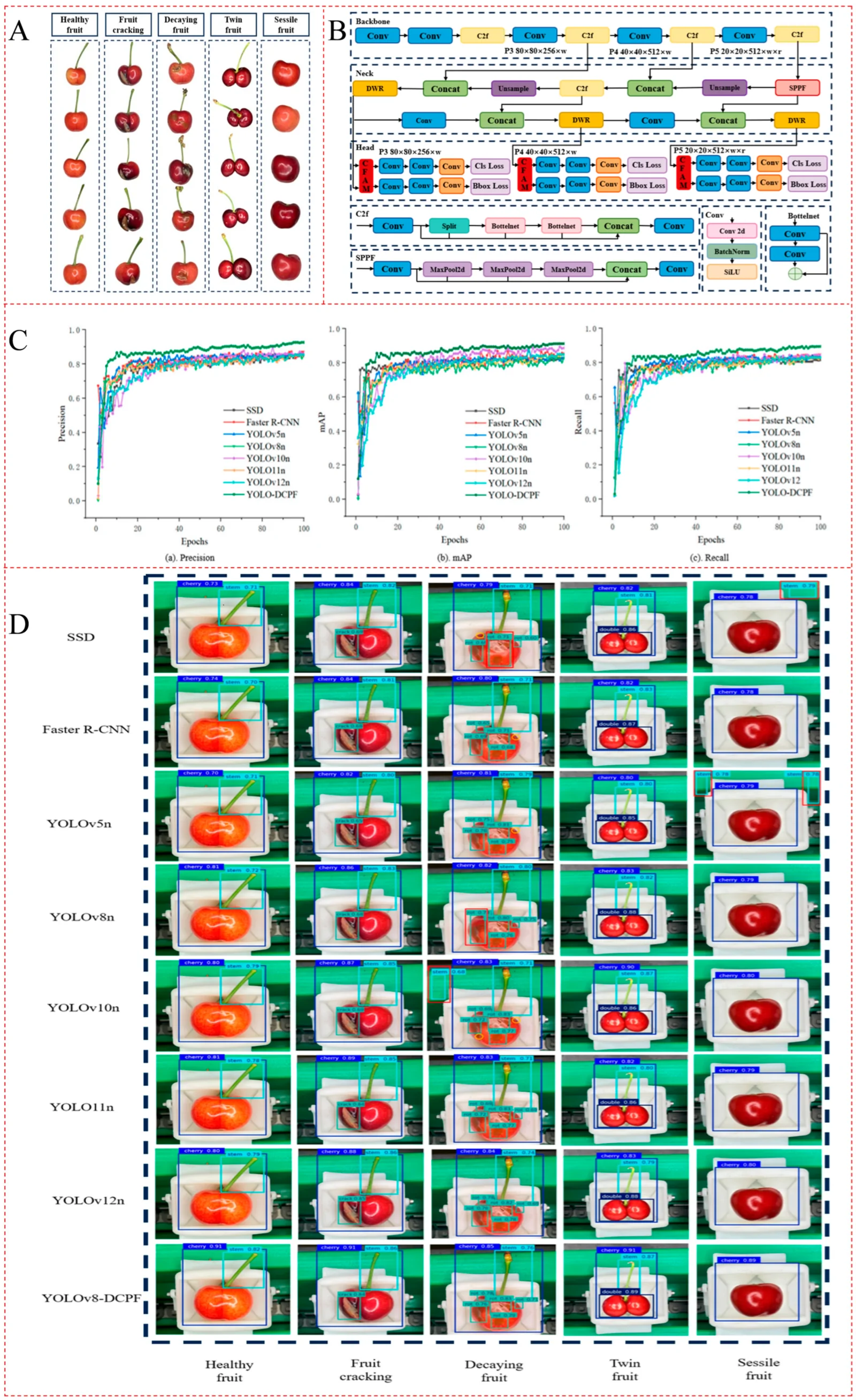
Representative applications in sweet-cherry grading. (**A**) Dataset images [121]. (**B**) YOLOv8-DCPF architecture [121]. (**C**) Precision, mean average precision and recall curves for the evaluated models [121]. (**D**) Test results of different models [121]. In (**B**), colored blocks distinguish the labeled network operations. In (**C**), line colors identify the models listed in each legend. In (**D**), colored boxes mark detections, with category labels and confidence scores printed beside the boxes.

The commercial grade of sweet cherries is determined primarily by cracking, decay, mechanical injury, and conjoined or stemless deformities. Defects occupy small areas, the fruit surface is highly reflective, and stems frequently cause occlusion; consequently, vision models require both fine-grained feature extraction and high-speed inference. Liu et al. proposed a YOLOv8-DCPF model incorporating DWR multiscale convolution, CAFM attention, PIoU loss, and self-distillation to identify sound, cracked, decayed, conjoined, and stemless malformed cherries. The model achieved a precision of 92.6%, mAP@0.5 of 91.2%, recall of 89.4%, and an F1-score of 89.0%, while occupying only 5.6 MB and processing 126 frames/s [121] (Figure 13A–D). This method is suitable for localizing small defects and rejecting shape abnormalities on continuous conveying lines. Commercial validation must nevertheless account for changes in stem orientation, duplicate detections caused by fruit rotation, and missed detection of small cracks on unobserved surfaces.

**Figure 14:**
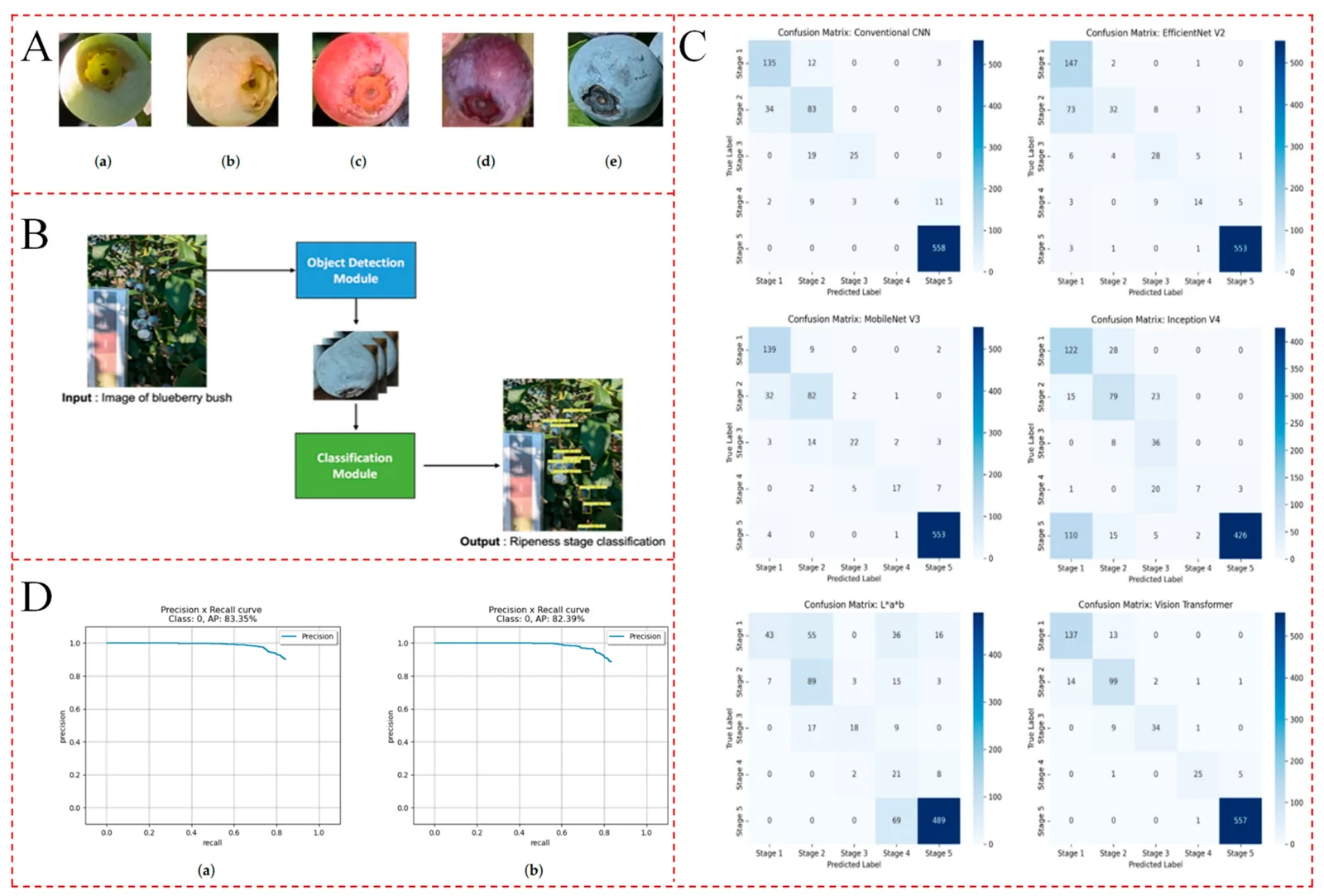
Representative applications in blueberry grading. (**A**) Dataset images showing (a) mature green, (b) green pink, (c) blue pink, (d) blue and (e) fully ripe fruit [20]. (**B**) Overview of the proposed method for the classification of the maturity level of blueberry fruit [20]. (**C**) Confusion matrix [20]. (**D**) Precision–recall curves for (a) object detection and (b) segmentation [20].

The principal challenges in blueberry grading are the dense arrangement of berries and the subtle color differences between adjacent ripening stages, while surface bloom and ambient illumination further alter fruit brightness. In addition, early bruising caused during harvesting and handling can damage tissues before visible symptoms develop. Esaki et al. acquired 250 orchard images at a resolution of 4032 × 3024 pixels and annotated 4506 blueberries. YOLOv8 was used to localize individual berries, and a Vision Transformer with 5.7 million parameters classified them into five stages: mature green, green-pink, blue-pink, blue, and fully ripe. The mean accuracy across three random dataset partitions was 93.7%, while the mean stage-specific accuracies were 88.8%, 83.1%, 84.7%, 71.9%, and 99.8%, respectively, identifying the transition from blue to fully ripe as the primary source of confusion [20] (Figure 14A–D). Liu et al. further fused hyperspectral, color, texture, and shape information and optimized SVM parameters using a peacock optimization algorithm. The resulting PKOA-SVM achieved recognition accuracies of 98.92% for stem-end bruising at 24 h and 94.56% for calyx-end bruising at 30 min. Compared with spectral features alone, texture–spectral fusion improved accuracy by 5.61 and 8.34 percentage points, respectively [122]. RGB vision is therefore appropriate for high-throughput preliminary maturity screening, whereas hyperspectral imaging is better reserved for berries with similar external appearances or suspected injury. When the two techniques are combined, low-drop rolling, compliant belt surfaces, or air-jet sorting should be used to preserve the surface bloom and minimize secondary bruising.

**Figure 15:**
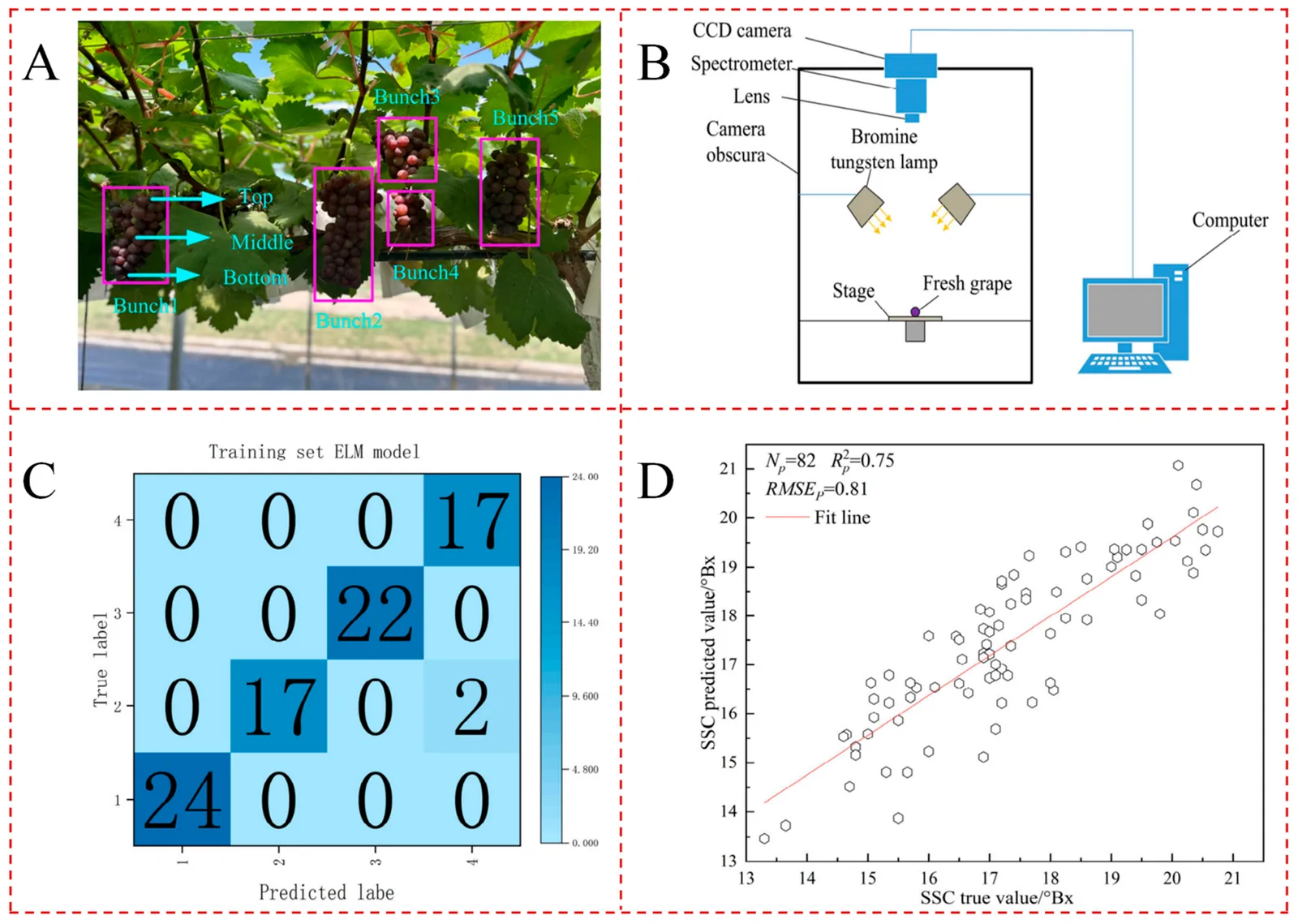
Representative applications in table-grape grading. (**A**) Sampled grape cluster [123]. (**B**) Hyperspectral imaging system [123]. (**C**) Confusion matrix for the extreme-learning-machine cultivar classifier [123]. (**D**) Predicted versus measured SSC for the raw-spectrum model [123].

Unlike cherries and blueberries, table grapes are generally graded as whole clusters. Overlapping berries cause visual occlusion and spectral scattering, while single-point measurements are highly sensitive to sampling position. Daniels et al. performed noncontact FT-NIR scans of 338 intact table-grape clusters and used PLS to simultaneously predict soluble solids content (SSC), titratable acidity (TA), the SSC/TA ratio, pH, and BrimA. The SSC model achieved R^2^p = 0.71 and RMSEP = 1.52 °Brix, while the BrimA model achieved R^2^p = 0.77 and RMSEP = 1.80 [21]. Chen et al. acquired hyperspectral images of four table-grape cultivars and employed a full-spectrum extreme learning machine for cultivar authentication and SSC prediction. The cultivar-classification accuracy reached 97.56%, while SSC prediction yielded R^2^p = 0.75 and RMSEP = 0.81 °Brix [123] (Figure 15A–D). These results indicate that whole-cluster or multipoint spectroscopy can support both cultivar authentication and internal-quality grading. Online implementation must nevertheless control cluster orientation, illumination distance, and effective surface coverage and should coordinate whole-cluster vision, spectroscopic maturity assessment, compliant weighing, and packaging specifications rather than directly applying models developed for individual berries.

Sweet cherries favor high-speed RGB defect screening, blueberries require small-target vision with selective latent-injury assessment, and table grapes require whole-cluster sensing. Across these commodities, evidence should report throughput, mild-defect recall, stem or surface-bloom damage, cross-season validation and post-grading shelf life.

## 7. Challenges and Prospects

Despite rapid technical advances, three deployment gaps persist: quality-to-grade mapping, model generalization under distribution shift, and low-damage system integration. Progress in closing these gaps should be evaluated against four commercial criteria: decision validity, continuous-operation reliability, fruit protection, and life-cycle value, rather than laboratory accuracy alone.

(1) Quality-to-grade mapping and selective sensor fusion. Commercial grading criteria should map measurable appearance traits, internal-quality attributes, and safety-related defects to specific market requirements, storage periods, transport distances, and loss-reduction targets. Machine vision should serve as the default high-throughput screening method. Vis/NIR, reduced-band imaging, or other modalities should be incorporated only when they alter an allocation decision. Sensor-fusion studies should evaluate each added modality against a single-sensor baseline in terms of incremental decision value, latency, and unit cost [43,53].
(2) Generalizable models and trustworthy online validation. Random data splits within a single batch provide evidence for model development but do not constitute independent validation of deployment readiness. A practical validation ladder progresses from held-out fruit in the same batch to an independent harvest batch, an unseen cultivar, season, or site, and finally prospective commercial-line testing. Only the final two levels support broad claims of model transferability. To improve generalization, multi-cultivar and multi-season datasets should be integrated with transfer learning, domain adaptation, online calibration, and continual model updating, alongside drift monitoring and version control [12,65]. Reports should quantify grade-specific recall, calibration or prediction error, inference latency, throughput, long-term drift, and misclassification costs.
(3) Low-damage integration and commercial maturity. Feeding, singulation, sensing, tracking, and actuation should be coordinated through a unified framework for timing and fruit identity. Complete-machine trials should report continuous operating time, jamming, cleaning and sanitation time, maintenance requirements, operator-training needs, packing-line compatibility, and energy use. They should also quantify fruit damage immediately and after 24–48 h, decay incidence, marketable yield, and return on investment [14,80]. Studies should report the technology readiness level or clearly distinguish among proof-of-concept, pilot-line, and validated commercial operation. Automated grading may complement trained inspectors through exception review in heterogeneous lots. Claims that it can fully replace human labor therefore require longitudinal evidence of stable quality, reduced losses, labor savings, and investment payback.

## 8. Conclusions

Postharvest fruit grading is shifting from mechanical sorting by size, mass and color toward integrated evaluation of external quality, internal attributes, latent defects and distribution suitability. Machine vision provides scalable external screening, whereas Vis/NIR and reduced-band spectral systems extend decisions to SSC, dry matter, firmness and internal disorders. The literature nevertheless shows that laboratory accuracy is not equivalent to commercial readiness: transfer across cultivars, seasons, sites and instruments, synchronized low-damage handling, sanitation, uptime and lifecycle economics remain decisive. Near-term deployment should therefore favor tiered, modular systems validated on independent production data and linked to storage, transport and market outcomes. Such systems are most credible when they complement human oversight for ambiguous cases and demonstrate stable loss reduction and return on investment over prolonged operation.

## Figures and Tables

**Table 1:** Representative studies and evidence maturity across sensing, algorithms, and complete equipment.

Fruit/Ref.	Method	Target	*n*	Mode	Validation	Performance/Speed	Key Limitation
Apple [5]	Multiview RGB + YOLOv5	External defects/grade	NR	Complete machine	Model test + machine trial	90.6% model; 93% machine; 4 fruit/s	Limited cross-cultivar and long-duration evidence
Apple [11]	Portable Vis/NIR + PLS	SSC	NR	Laboratory and field	Independent laboratory; field recalibration	R^2^ = 0.764 (laboratory); 0.690 (field)	Environment- and batch-specific calibration
Apple [12]	BiSeNet V2 + pruned YOLOv4	Defect area/grade	NR	Online system	Internal test + online grading	92.42% online; 9 ms/image	Independent production-line validation not reported
Apple [13]	Three-channel helical conveyor	Singulation/full-surface view	NR	Pilot line	Bench-line trial	>9 fruit/s total	Surface coverage decreases as channel speed increases
Orange [14]	RGB detector + SORT tracking	Mechanical damage/lesions	300; 2400 images	Online video	Recorded-line evaluation	93.66%; 43 frames/s	Actuator conceptual; edge fruit may be incompletely viewed
Fresh jujube [15]	Vision + electromagnetic actuation	Size/color grade	NR	Complete machine	Machine trial	93.7%; 20 fruit/s; 1400 kg/h	Single-machine evidence; long-term drift not reported
Apple [16]	Dynamic Vis/NIR transmittance	Moldy core	340 fruit	Online sensing	Prediction set	AUC = 0.99; 98.82% binary accuracy	Single cultivar; throughput not reported
Peach [17]	Multispectral structured illumination	Early slight bruising	300 fruit	Static acquisition	Controlled-impact dataset	96% accuracy	Static system; sutures and lenticels confound segmentation
Apricot [18]	Portable Vis/NIR + HSI	SSC, dry matter, acidity	204; 17 cv.	Offline	Two years; four external cultivars	HSI R^2^ = 0.904, 0.918 and 0.811	Characteristic-band reduction needed for online use
Citrus [19]	Four-path Vis/NIR transmittance	Fruit-fly infestation	252 fruit	Offline bench	Internal train/test across orientations	Overall 89.3–92.9%; mild class 80.1%	Multipath complexity and weak mild-infestation recall
Blueberry [20]	YOLOv8 + Vision Transformer	Five maturity stages	250 images; 4506 berries	Orchard images	Three random splits	93.7% mean accuracy	Not independent production validation; blue stage 71.9%
Table grape [21]	Noncontact FT-NIR + PLS	SSC and maturity indices	338 clusters	Offline	Prediction set	SSC R^2^p = 0.71; RMSEP = 1.52 °Brix	Cluster occlusion and sampling-position sensitivity

Note: NR, not reported in the reviewed manuscript text; HSI, hyperspectral imaging. An internal random split is not considered equivalent to an independent production-batch or commercial-line validation. Representative studies met the coverage and scale or operational criteria defined in Review Methodology and Supplementary Methods S1; formal domain scores are provided in Supplementary Table S2 (Supplementary Materials).

**Table 2:** Comparison of key technologies for postharvest fruit grading.

Technology	Core Information	Advantages	Applications	Limitations	References
Machine vision	Size, shape, color, texture, and surface defects	High throughput, low cost, and easy integration	External grading and defect rejection	Limited internal-quality assessment; sensitive to illumination, occlusion, and domain shift	[26]
Visible-near-infrared spectroscopy	SSC, dry matter, firmness, moisture, and maturity	Rapid and nondestructive; suitable for handheld or online use	Internal-quality grading and postharvest decision-making	Local sampling; difficult calibration across cultivars or instruments	[11]
Hyperspectral imaging	Spatial information and continuous spectra	Localization of latent defects and visualization of quality distributions	Early-damage detection and informative-wavelength selection	High cost and data volume; complex online calibration	[29]
X-ray imaging	Tissue density and internal structure	Strong penetration and visualization of internal defects	Precision internal inspection and quarantine	High cost and safety requirements; limited CT throughput	[41]
Acoustic/mechanical sensing	Resonance, impact, and compression responses	Simple structure; sensitive to firmness and maturity	Texture and maturity grading	Requires controlled excitation/contact; sensitive to orientation and noise	[42]
Electronic nose	Volatile-organic-compound fingerprints	Responsive to maturity, injury, and microbial metabolism	Decay warning and aroma monitoring	Susceptible to environmental interference and drift; slow recovery	[30]
Multisensor fusion	Appearance, composition, structure, and odor information	Complementary multi-attribute information and greater robustness	Comprehensive quality and shelf-life grading	Complex synchronization, registration, and missing-modality handling; high cost	[43]

**Table 3:** Comparison of conventional machine-learning algorithms.

Algorithm	Primary Function/Input	Advantages	Limitations	Suitable Applications	References
PCA	Dimensionality reduction and feature compression	Unsupervised and rapid; reduces collinearity	Principal components may not be associated with grade	Preprocessing of spectra or multi-feature data	[56]
PLS/PLS-DA	Spectral regression or classification	Suitable for high-dimensional collinear data; interpretable	Sensitive to nonlinearity and domain shift	Prediction of SSC, firmness, and maturity	[57]
K-means	Unsupervised clustering and initial grade partitioning	No labels required; computationally simple	Requires a predefined number of clusters; sensitive to initialization and scale	Exploratory grading with clear grade boundaries	[58]
SVM/SVR	Small-sample classification or nonlinear regression	Good generalization for high-dimensional, small-sample data	Sensitive to kernel and hyperparameters; slow on large datasets	Image-feature classification and spectral prediction	[59]
Random forest	Nonlinear classification/regression and variable ranking	Resistant to overfitting; handles mixed features	Relatively large model; limited extrapolation	Multisource feature modeling and selection	[60]
KNN	Neighborhood-distance classification	No training required; simple implementation	Slow prediction; sensitive to scale and noise	Benchmark classification for small datasets	[61]

**Table 4:** Comparison of deep-learning algorithms.

Algorithm	Primary Function/Input	Advantages	Limitations	Suitable Applications	References
CNN classifier	Images/grade labels	Automatic feature learning; mature workflow	Does not localize defects; high annotation demand	Maturity, cultivar, and defect classification	[68]
YOLO/SSD	One-stage fruit/defect localization	End-to-end, rapid, and readily deployable	Prone to missing small defects and occluded targets	High-speed online multi-fruit detection	[62]
Faster R-CNN	Two-stage detection based on region proposals	High localization accuracy; adaptable to complex targets	Large model and slow inference	Offline precision inspection or low-speed applications	[69]
U-Net/DeepLab/BiSeNet	Pixel-level fruit/defect segmentation	Quantifies defect area, shape, and proportion	Expensive pixel-level annotation; sensitive to boundary error	Refined defect grading	[12]
Vision Transformer	Attention-based global relationship modeling	Strong global representation; suitable for multimodal data	High data and computing requirements; susceptible to overfitting on small datasets	Complex scenes and fusion tasks	[65]

**Table 5:** Comparison of model-transfer, lightweight, and deployment methods.

Method	Primary Function/Input	Advantages	Limitations	Suitable Applications	References
Transfer learning/fine-tuning	Pretrained parameters plus a small target-domain dataset	Reduces annotation and training costs; improves small-sample performance	Large domain differences may cause negative transfer	New cultivars, production regions, or defect tasks	[66]
Calibration transfer/domain adaptation	Alignment of instrument, seasonal, or batch distributions	Extends the useful life of spectral models	Requires target-domain samples; performance depends on the type of domain shift	Quality models across seasons or instruments	[73]
Model pruning	Removal of redundant channels, layers, or weights	Reduces model size and inference latency	Excessive pruning may remove small-target information	Edge devices and high-speed detection	[12]
Parameter quantization	Reduction in weight and activation precision	Reduces storage, bandwidth, and energy consumption	May reduce accuracy; depends on hardware support	INT8/FP16 edge inference	[71]
Knowledge distillation	Teacher-model supervision of a student model	Retains high accuracy in a small model	Complex training; depends on teacher quality	High-accuracy lightweight networks	[69]
Edge deployment and acceleration	Model compilation, operator optimization, and hardware inference	Reduces end-to-end latency	Platform-dependent; requires joint tuning of acquisition and control	Industrial computers, Jetson devices, and embedded terminals	[72]

**Table 6:** Representative mechanisms in feeding and cleaning units.

Mechanism	Main Components	Function and Advantages	Suitable Fruit and Design Considerations	References
Hopper plus elevator belt	Cushioning hopper, elevator belt, and metering gate	Continuous, metered feeding	Medium-to-large fruit; control drop height and bridging	[5]
Water flume/hydraulic conveying	Cushioning tank, circulation pump, and guide channel	Gentle receiving with simultaneous cleaning	Damage-sensitive fruit; control flow rate, water quality, and cross-contamination	[2]
Spraying plus brush rollers	Nozzles, brush rollers, and recirculating filtration	Continuous removal of soil and adhering matter	Apples and citrus fruit; optimize brush stiffness, rotational speed, and spray pressure	[76]
Ultrasonically assisted cleaning	Cleaning tank, transducers, and generator	Enhanced removal of microorganisms and residues	Tolerant fruit; limit power to prevent tissue damage	[75]
Air-knife/cool-air drying	Blower, air knife, and drainage section	Removes surface water and stabilizes optical inspection	All fruit types; avoid heating and water loss	[77]

**Table 7:** Representative mechanisms for fruit singulation, conveying, and orientation.

Mechanism	Motion Pattern	Primary Advantages	Limitations and Suitable Applications	References
Differential-speed conveyor belts	Progressive separation using multiple belt speeds	Compact structure and high throughput	No active rotation; suitable for regular, damage-tolerant fruit	[78]
Helical/double-cone rollers	Simultaneous separation and rotation during conveying	Extensive surface coverage; supports multiview imaging	Sensitive to fruit diameter and roller gap; suitable for apples, citrus fruit, and pears	[13]
Fruit cups/trays	Individual support, coded positioning, and point-specific discharge	Stable position; readily integrates weighing and diversion	Limited rotation; suitable for standardized grading lines	[5]
Brush/compliant rollers	Slow rotation driven by compliant contact	Low damage; can also support cleaning	Limited orientation consistency; suitable for peaches and kiwifruit	[6]
Suspended-grip conveying	Clamp-supported, suspended transport	Minimal contact and flexible optical-path arrangement	Complex gripping and release; suitable for strawberries and grapes	[81]

**Table 8:** Representative sensor configurations for online inspection.

Inspection Method	Key Hardware	Measurable Attributes	Suitable Applications and Engineering Considerations	References
Machine vision	Industrial camera, lens, illumination, and light-shielding enclosure	Size, shape, color, and surface defects	High-speed external grading; control reflection, shadows, and surface coverage	[89]
Visible-near-infrared spectroscopy	Light source, optical fiber, spectrometer, and light-shielding structure	SSC, dry matter, maturity, and internal defects	Internal-quality grading; stabilize optical path, temperature, and sampling position	[82]
Hyper/multispectral imaging	Line-scan camera, scanning mechanism, and broadband illumination	Spatial distribution plus continuous spectra	Precision defect inspection; requires dimensionality reduction and wavelength selection	[85]
X-ray imaging	X-ray source, detector, and safety interlock	Cavities, insect damage, cracks, and structural abnormalities	High-value/safety screening; balance protection, cost, and throughput	[83]
Weighing/mechanical sensing	Weighing cup and pressure, impact, or vibration sensors	Mass, firmness, and impact response	Size grading and multisource assessment; requires dynamic compensation and periodic calibration	[79]

**Table 9:** Representative architectures for intelligent classification and control.

Control Architecture	Main Components	Advantages	Suitable Applications and Considerations	References
Threshold rules plus PLC	Sensors, PLC, encoder, and I/O	Highly deterministic, low cost, and easy maintenance	Rule-based size/mass grading; limited representation of complex defects	[93]
Industrial computer plus PLC	Industrial computer/GPU, PLC, and camera/spectrometer	Combines complex inference with reliable motion control	Multisensor production lines; requires communication-latency compensation	[12]
Edge AI plus microcontroller	Jetson/AI chip, MCU/PLC, and fieldbus	Compact, low latency, and readily modularized	Limited by computing capacity, heat dissipation, and quantization error	[69]
Detection and tracking plus encoder	Detection/tracking, fruit IDs, encoder, and buffer	Accurate mapping between inspection and actuation stations	Continuous multi-fruit handling; must address dropped frames, occlusion, and speed variation	[14]
Multisensor decision fusion	Synchronized acquisition, fusion model, and timing/calibration module	Joint assessment of external and internal quality	Comprehensive grading; high synchronization, calibration, and maintenance costs	[86]

**Table 10:** Representative sorting actuators.

Actuation Method	Typical Components	Advantages	Limitations and Suitable Fruit	References
Air-jet diversion	Compressed air, solenoid valve, and nozzle	High-speed, noncontact operation, and simple structure	Sensitive to mass and orientation; suitable for small, light fruit or rapid rejection	[8]
Paddle/pusher diversion	Motor/cylinder, paddle/pusher plate, and cushioned channel	Low cost and direct control	Relatively high contact impact; suitable for apples and citrus fruit	[15]
Flap/gate diversion	Flap/bottom gate, guide plate, and actuator	Clear grade-to-channel mapping; supports multiple channels	Requires precise timing and reset; suitable for cup-based grading lines	[5]
Conveyor-lane diversion	Guide channels, diverter belts, and multistage chutes	Smooth diversion and easy linkage to packaging	Large footprint; suitable for medium-to-large commercial lines	[98]
Robotic compliant gripping	Robotic arm, soft fingers/suction cup, and force/slip sensors	High flexibility, low damage, and point-specific placement	High cost and slow cycle; suitable for high-value, damage-sensitive fruit	[80]

## Data Availability

No new data were created or analyzed in this study. Data sharing is not applicable to this article.

## Supplementary Materials

The following supporting information can be downloaded at: https://www.mdpi.com/article/10.3390/foods15183308/s1, Supplementary Methods S1. Evidence selection and maturity appraisal. Table S1. Operational evidence-maturity criteria and thresholds. Table S2. Item-level appraisal of the representative studies reported in Table 1. Table S3. Evidence-role map for all references included in the review.

## Abbreviations

The following abbreviations are used in this manuscript:

1D-CNN	One-dimensional convolutional neural network
ANN	Artificial neural network
AUC	Area under the receiver operating characteristic curve
BiSeNet	Bilateral segmentation network
BPNN	Backpropagation neural network
CARS	Competitive adaptive reweighted sampling
CNN	Convolutional neural network
CT	Computed tomography
Faster R-CNN	Faster region-based convolutional neural network
FLOPs	Floating-point operations
FT-NIR	Fourier-transform near-infrared
GA	Genetic algorithm
GPU	Graphics processing unit
HSI	Hyperspectral imaging
ICA	Independent component analysis
IoU	Intersection over union
KNN	K-nearest neighbors
LDA	Linear discriminant analysis
LDV	Laser doppler vibrometry
LightGBM	Light gradient boosting machine
LS-SVM	Least squares support vector machine
LSTM	Long short-term memory
mAP	Mean average precision
MC-UVE	Monte carlo uninformative variable elimination
MCU	Microcontroller unit
MLP	Multilayer perceptron
MSIRI	Multispectral structured-illumination reflectance imaging
NIR	Near-infrared
PCA	Principal component analysis
PLC	Programmable logic controller
PLS	Partial least squares
PLS-DA	Partial least squares discriminant analysis
R^2^	Coefficient of determination
RF	Random forest
RMSEP	Root mean square error of prediction
RPD	Residual predictive deviation
SPA	Successive projections algorithm
SRM	Singulating and rotating mechanism
SSC	Soluble solids content
SSD	Single shot multibox detector
SVM	Support vector machine
SVR	Support vector regression
TA	Titratable acidity
TSS	Total soluble solids
ViT	Vision transformer
Vis/NIR	Visible-near-infrared
WOA	Whale optimization algorithm
XGBoost	Extreme gradient boosting
YOLO	You only look once
