# Supplementary material for: Postharvest Fruit Grading Technologies and Equipment: A Review
Source: Foods. 2026 Sep 18;15(18):3308. doi: 10.3390/foods15183308 (PMC13606810; doi:10.3390/foods15183308)
Supplement: Supplementary file 1 [file foods-15-03308-s001.zip › foods-4524801-supplementary.pdf]

# Supplementary Materials for

# Postharvest Fruit Grading Technologies and Equipment: A Review

**Jianli Hu [1], Lixin Ma [1,2], Wenya Zhang [3], Jinxiu Song [1] and Pengpeng Yu [1,*]**

## Supplementary Methods S1. Evidence selection and maturity appraisal

Purpose and scope. The framework separates technical feasibility from evidence for transferability and commercial deployment. It was applied to primary empirical studies selected as representative examples in Table 1. Secondary reviews and contextual engineering or biological sources were mapped by role in Table S3 but were not assigned an empirical maturity score.

Representative-study selection for Table 1. A study had to address a distinct sensing modality, algorithm, equipment configuration or fruit-handling context, report at least one quantitative performance outcome, and meet at least one benchmark: at least 200 individual fruit or fruit clusters, at least 2000 labelled images or fruit instances, at least 4 fruit $s^{-1}$, or at least 25 frames $s^{-1}$. A study below these thresholds could still be selected if it included independent validation across batches, cultivars, seasons or sites, or an online, pilot-line or commercial-line trial. These thresholds are operational rules for this review and are not universal definitions of adequate study size or industrial performance.

Coding. Six domains were scored from 0 to 2. NR denoted information not reported in the reviewed source and received 0 points. Exact threshold values were assigned to the higher bin, while ambiguous or borderline cases were assigned to the lower bin. Any coding disagreement was resolved by rechecking the source against the prespecified definitions. If the higher category remained unsupported, the value was coded NR or retained in the lower category. Scores describe evidence maturity rather than intrinsic scientific merit.

Synthesis weighting. Totals of 0–3 were classified as limited, 4–6 as moderate, 7–9 as strong within the tested context, and 10–12 as deployment-level. Limited evidence was used only to support feasibility. Moderate evidence supported comparisons within the tested commodity and setting. Strong evidence could inform transferability or operational conclusions only within the validated scope. Deployment-level interpretation additionally required a validation score of 2 and an operational-setting score of 2, so sample scale or processing speed alone could not establish commercial maturity.

**Table S1.** Operational evidence-maturity criteria and thresholds.

| Domain | 0 points | 1 point | 2 points | Interpretive safeguard |
|---|---|---|---|---|
| Sample scale | NR, <100 fruit/clusters and <1000 labelled images/instances | 100–199 fruit/clusters or 1000–1999 images/instances | ≥200 fruit/clusters or ≥2000 images/instances | Image and fruit counts are recorded separately; image-based credit requires a fruit-disjoint or otherwise independent unit of analysis. |
| Biological/environmental diversity | Single cultivar/batch/season/site or NR | Variation in one axis, such as cultivar, batch, season or site | Variation in at least two axes, or explicit multi-year plus external-cultivar/site coverage | Diversity earns credit only when linked to calibration or validation, not merely mentioned. |

| | | | | |
|---|---|---|---|---|
| Validation independence | Training-only, resubstitution, internal random split or NR | Held-out prediction set, controlled independent test, or same-source machine/recorded-line trial | Independent batch, cultivar, season or site test, or prospective commercial-line validation | Internal random splitting cannot receive 2 points. |
| Operational setting | Offline laboratory/static acquisition or NR | Online sensor, recorded line, bench line, pilot line or complete-machine trial | Prospective routine commercial-line operation | A portable instrument without line testing remains 0 unless operational use is documented. |
| Throughput | NR | Reported but <4 fruit s−1 or <25 frames s−1 | ≥4 fruit s−1 or ≥25 frames s−1 | Frame rate is not treated as fruit throughput; both units remain explicit. |
| Reproducibility/reporting | Key protocol, validation partition or quantitative result not reported | Method, quantitative result and main limitation are reported | Repeated-run uncertainty plus accessible data/code or a sufficiently detailed public protocol | No credit is inferred from journal prestige or model complexity. |

**Table S2. Item-level appraisal of the representative studies reported in Table 1.**

Scores were assigned conservatively from the information summarized in the revised manuscript and Table 1. S, sample scale; D, diversity; V, validation independence; O, operational setting; T, throughput; R, reproducibility/reporting; NR, not reported.

| Ref. | Fruit | Method | S | D | V | O | T | R | Total | Category | Basis and limitation |
|---|---|---|---|---|---|---|---|---|---|---|---|
| [5] | Apple | Multiview RGB + YOLOv5 | 0 | 0 | 1 | 1 | 2 | 1 | 5 | Moderate | Machine trial and 4 fruit s−1; sample scale and diversity were NR. |
| [11] | Apple | Portable Vis/NIR + PLS | 0 | 0 | 2 | 0 | 0 | 1 | 3 | Limited | Independent laboratory validation and field recalibration; sample scale and throughput were NR. |
| [12] | Apple | BiSeNet V2 + pruned YOLOv4 | 0 | 0 | 1 | 1 | 2 | 1 | 5 | Moderate | Online grading and 9 ms image−1; independent production-line validation was not reported. |
| [13] | Apple | Three-channel helical conveyor | 0 | 0 | 1 | 1 | 2 | 1 | 5 | Moderate | Bench-line trial and more than 9 fruit s−1; sample scale and diversity were NR. |
| [14] | Orange | RGB detector + SORT tracking | 2 | 0 | 1 | 1 | 2 | 1 | 7 | Strong within tested context | 300 fruit and 2400 images; recorded-line evaluation at 43 frames s−1; actuator remained conceptual. |
| [15] | Fresh jujube | Vision + electromagnetic actuation | 0 | 0 | 1 | 1 | 2 | 1 | 5 | Moderate | Complete-machine trial at 20 fruit s−1; sample scale and long-term drift were NR. |

| | | | | | | | | | | |
|---|---|---|---|---|---|---|---|---|---|---|
| [16] | Apple | Dynamic Vis/NIR transmittance | 2 | 0 | 1 | 1 | 0 | 1 | 5 | Moderate | 340 fruit and an online sensing configuration; single cultivar and throughput NR. |
| [17] | Peach | Multispectral structured illumination | 2 | 0 | 1 | 0 | 0 | 1 | 4 | Moderate | 300 fruit with a controlled-impact dataset; static acquisition and no throughput report. |
| [18] | Apricot | Portable Vis/NIR + HSI | 2 | 2 | 2 | 0 | 0 | 1 | 7 | Strong within tested context | 204 fruit, 17 cultivars, two years, and four external cultivars; offline acquisition. |
| [19] | Citrus | Four-path Vis/NIR transmittance | 2 | 0 | 0 | 0 | 0 | 1 | 3 | Limited | 252 fruit; internal train/test evaluation, offline bench setting, and no throughput report. |
| [20] | Blueberry | YOLOv8 + Vision Transformer | 2 | 0 | 0 | 0 | 0 | 1 | 3 | Limited | 4506 berries in 250 images; three random splits and no independent production validation. |
| [21] | Table grape | Noncontact FT-NIR + PLS | 2 | 0 | 1 | 0 | 0 | 1 | 4 | Moderate | 338 clusters and a prediction set; offline testing and no throughput report. |

**Table S3. Evidence-role map for all references included in the review.**

This table accounts for the complete reference list. Formal six-domain scores are reported in Table S2 for the representative empirical exemplars used in Table 1. Other primary studies contribute to modality- or commodity-specific narrative synthesis; secondary and contextual sources are not treated as empirical deployment evidence. The map contains 95 primary empirical studies, including 12 scored exemplars, 20 secondary or contextual synthesis sources, and 8 handling or mechanism studies.

| Ref. | Year | Title | Evidence role | Appraisal disposition and use in synthesis |
|---|---|---|---|---|
| [1] | 2017 | Postharvest losses of fruit and vegetables during retail and in consumers' homes: Quantifications, causes, and means of prevention | Secondary review or contextual synthesis | Mapped for context, terminology or prior synthesis; not assigned an empirical maturity score and not used as direct deployment evidence. |
| [2] | 2022 | Mechanical damages and packaging methods along the fresh fruit supply chain: A review | Secondary review or contextual synthesis | Mapped for context, terminology or prior synthesis; not assigned an empirical maturity score and not used as direct deployment evidence. |
| [3] | 2022 | Fruit quality evaluation using machine learning techniques: review, motivation and future perspectives | Secondary review or contextual synthesis | Mapped for context, terminology or prior synthesis; not assigned an empirical maturity score and not used as direct deployment evidence. |
| [4] | 2025 | Artificial Vision Systems for Fruit Inspection and Classification: Systematic Literature Review | Secondary review or contextual synthesis | Mapped for context, terminology or prior synthesis; not assigned an empirical maturity score and not used as direct deployment evidence. |
| [5] | 2023 | Apple Grading Method Design and Implementation for Automatic Grader Based on Improved YOLOv5 | Representative primary empirical study | Formally scored in Table S2; used with the synthesis weight permitted by its maturity category and validation scope. |

| | | | | |
|---|---|---|---|---|
| [6] | 2023 | Apple Grading Based on Multi-Dimensional View Processing and Deep Learning | Primary empirical study in narrative synthesis | Used for modality- or commodity-specific findings; no broad transfer or deployment claim was assigned unless independent or online evidence was explicitly reported. |
| [7] | 2025 | Non-Destructive Detection of Fruit Quality: Technologies, Applications and Prospects | Secondary review or contextual synthesis | Mapped for context, terminology or prior synthesis; not assigned an empirical maturity score and not used as direct deployment evidence. |
| [8] | 2020 | Development of near-infrared online grading device for long jujube | Primary empirical study in narrative synthesis | Used for modality- or commodity-specific findings; no broad transfer or deployment claim was assigned unless independent or online evidence was explicitly reported. |
| [9] | 2022 | Two-wavelength image detection of early decayed oranges by coupling spectral classification with image processing | Primary empirical study in narrative synthesis | Used for modality- or commodity-specific findings; no broad transfer or deployment claim was assigned unless independent or online evidence was explicitly reported. |
| [10] | 2023 | Development of an optimally designed real-time automatic citrus fruit grading-sorting machine leveraging computer vision-based adaptive deep learning model | Primary empirical study in narrative synthesis | Used for modality- or commodity-specific findings; no broad transfer or deployment claim was assigned unless independent or online evidence was explicitly reported. |
| [22] | 2017 | Towards a new definition of quality for fresh fruits and vegetables | Secondary review or contextual synthesis | Mapped for context, terminology or prior synthesis; not assigned an empirical maturity score and not used as direct deployment evidence. |
| [23] | 2020 | Visible-NIR 'point' spectroscopy in postharvest fruit and vegetable assessment: The science behind three decades of commercial use | Secondary review or contextual synthesis | Mapped for context, terminology or prior synthesis; not assigned an empirical maturity score and not used as direct deployment evidence. |

| | | | | |
|---|---|---|---|---|
| [24] | 2021 | Nondestructive detection of decayed blueberry based on information fusion of hyperspectral imaging (HSI) and low-Field nuclear magnetic resonance (LF-NMR) | Primary empirical study in narrative synthesis | Used for modality- or commodity-specific findings; no broad transfer or deployment claim was assigned unless independent or online evidence was explicitly reported. |
| [25] | 2022 | Mechanical damage of fresh produce in postharvest transportation: Current status and future prospects | Secondary review or contextual synthesis | Mapped for context, terminology or prior synthesis; not assigned an empirical maturity score and not used as direct deployment evidence. |
| [26] | 2021 | Real-time visual inspection system for grading fruits using computer vision and deep learning techniques | Primary empirical study in narrative synthesis | Used for modality- or commodity-specific findings; no broad transfer or deployment claim was assigned unless independent or online evidence was explicitly reported. |
| [27] | 2025 | Real-time banana freshness grading: A portable end-to-end detection system with high precision | Primary empirical study in narrative synthesis | Used for modality- or commodity-specific findings; no broad transfer or deployment claim was assigned unless independent or online evidence was explicitly reported. |
| [32] | 2024 | Classification of Apple Color and Deformity Using Machine Vision Combined with CNN | Primary empirical study in narrative synthesis | Used for modality- or commodity-specific findings; no broad transfer or deployment claim was assigned unless independent or online evidence was explicitly reported. |
| [33] | 2025 | Strawberry Fruit Deformity Detection and Symmetry Quantification Using Deep Learning and Geometric Feature Analysis | Primary empirical study in narrative synthesis | Used for modality- or commodity-specific findings; no broad transfer or deployment claim was assigned unless independent or online evidence was explicitly reported. |
| [34] | 2019 | Grading bunch tightness for grape by multiperspective imaging approach coupled with multivariate classification methods | Primary empirical study in narrative synthesis | Used for modality- or commodity-specific findings; no broad transfer or deployment claim was assigned unless independent or online evidence was explicitly reported. |
| [35] | 2018 | New approach of simultaneous, multi-perspective imaging for quantitative assessment of the compactness of grape bunches | Primary empirical study in narrative synthesis | Used for modality- or commodity-specific findings; no broad transfer or deployment claim was assigned unless independent or online evidence was explicitly reported. |

| | | | | |
|---|---|---|---|---|
| [36] | 2025 | Apple maturity quantification based on fine-grained coloration analysis using deep learning and computer vision | Primary empirical study in narrative synthesis | Used for modality- or commodity-specific findings; no broad transfer or deployment claim was assigned unless independent or online evidence was explicitly reported. |
| [37] | 2025 | Identification of apple variety using machine vision and deep learning with Multi-Head Attention mechanism and GLCM | Primary empirical study in narrative synthesis | Used for modality- or commodity-specific findings; no broad transfer or deployment claim was assigned unless independent or online evidence was explicitly reported. |
| [38] | 2025 | Detection of early decay in dekopon fruit based on structured-illumination reflectance imaging combined with a comparison between traditional machine learning and deep learning models | Primary empirical study in narrative synthesis | Used for modality- or commodity-specific findings; no broad transfer or deployment claim was assigned unless independent or online evidence was explicitly reported. |
| [39] | 2024 | Developing universal classification models for the detection of early decayed citrus by structured-illumination reflectance imaging coupling with deep learning methods | Primary empirical study in narrative synthesis | Used for modality- or commodity-specific findings; no broad transfer or deployment claim was assigned unless independent or online evidence was explicitly reported. |
| [40] | 2025 | Transforming Apple Grading: Standards Survey and Deep Learning Insights | Secondary review or contextual synthesis | Mapped for context, terminology or prior synthesis; not assigned an empirical maturity score and not used as direct deployment evidence. |
| [44] | 2020 | Accurate non-destructive prediction of peach fruit internal quality and physiological maturity with a single scan using near infrared spectroscopy | Primary empirical study in narrative synthesis | Used for modality- or commodity-specific findings; no broad transfer or deployment claim was assigned unless independent or online evidence was explicitly reported. |
| [11] | 2020 | Non-destructive evaluation of soluble solids content of apples using a developed portable Vis/NIR device | Representative primary empirical study | Formally scored in Table S2; used with the synthesis weight permitted by its maturity category and validation scope. |

| | | | | |
|---|---|---|---|---|
| [45] | 2022 | General model of multi-quality detection for apple from different origins by Vis/NIR transmittance spectroscopy | Primary empirical study in narrative synthesis | Used for modality- or commodity-specific findings; no broad transfer or deployment claim was assigned unless independent or online evidence was explicitly reported. |
| [28] | 2020 | Nondestructive monitoring storage quality of apples at different temperatures by near-infrared transmittance spectroscopy | Primary empirical study in narrative synthesis | Used for modality- or commodity-specific findings; no broad transfer or deployment claim was assigned unless independent or online evidence was explicitly reported. |
| [46] | 2020 | Quantitative detection of apple watercore and soluble solids content by near infrared transmittance spectroscopy | Primary empirical study in narrative synthesis | Used for modality- or commodity-specific findings; no broad transfer or deployment claim was assigned unless independent or online evidence was explicitly reported. |
| [29] | 2023 | Detection of early bruises on apples using hyperspectral reflectance imaging coupled with optimal wavelengths selection and improved watershed segmentation algorithm | Primary empirical study in narrative synthesis | Used for modality- or commodity-specific findings; no broad transfer or deployment claim was assigned unless independent or online evidence was explicitly reported. |
| [47] | 2024 | Deep learning algorithm development for early detection of Botrytis cinerea infected strawberry fruit using hyperspectral fluorescence imaging | Primary empirical study in narrative synthesis | Used for modality- or commodity-specific findings; no broad transfer or deployment claim was assigned unless independent or online evidence was explicitly reported. |
| [48] | 2022 | Detection of soluble solid content in apples based on hyperspectral technology combined with deep learning algorithm | Primary empirical study in narrative synthesis | Used for modality- or commodity-specific findings; no broad transfer or deployment claim was assigned unless independent or online evidence was explicitly reported. |
| [49] | 2023 | Non-destructive prediction of total soluble solids and titratable acidity in Kyoho grape using hyperspectral imaging and deep learning algorithm | Primary empirical study in narrative synthesis | Used for modality- or commodity-specific findings; no broad transfer or deployment claim was assigned unless independent or online evidence was explicitly reported. |

| | | | | |
|---|---|---|---|---|
| [50] | 2025 | Automatic early bruise detection in strawberry fruit by hyperspectral imaging and deep learning techniques | Primary empirical study in narrative synthesis | Used for modality- or commodity-specific findings; no broad transfer or deployment claim was assigned unless independent or online evidence was explicitly reported. |
| [41] | 2023 | BraeNet: Internal disorder detection in 'Braeburn' apple using X-ray imaging data | Primary empirical study in narrative synthesis | Used for modality- or commodity-specific findings; no broad transfer or deployment claim was assigned unless independent or online evidence was explicitly reported. |
| [42] | 2020 | Nondestructive measurement of kiwifruit firmness, soluble solid content (SSC), titratable acidity (TA), and sensory quality by vibration spectrum | Primary empirical study in narrative synthesis | Used for modality- or commodity-specific findings; no broad transfer or deployment claim was assigned unless independent or online evidence was explicitly reported. |
| [51] | 2024 | Assessment of apple bruise resistance under transient collisions through X-ray computed tomography and image processing | Primary empirical study in narrative synthesis | Used for modality- or commodity-specific findings; no broad transfer or deployment claim was assigned unless independent or online evidence was explicitly reported. |
| [30] | 2021 | Identification of the apple spoilage causative fungi and prediction of the spoilage degree using electronic nose | Primary empirical study in narrative synthesis | Used for modality- or commodity-specific findings; no broad transfer or deployment claim was assigned unless independent or online evidence was explicitly reported. |
| [52] | 2023 | Spoilage Monitoring and Early Warning for Apples in Storage Using Gas Sensors and Chemometrics | Primary empirical study in narrative synthesis | Used for modality- or commodity-specific findings; no broad transfer or deployment claim was assigned unless independent or online evidence was explicitly reported. |
| [43] | 2024 | Research on prediction of yellow flesh peach firmness using a novel acoustic real-time detection device and Vis/NIR technology | Primary empirical study in narrative synthesis | Used for modality- or commodity-specific findings; no broad transfer or deployment claim was assigned unless independent or online evidence was explicitly reported. |
| [53] | 2021 | Classification of Apples Based on the Shelf Life Using ANN and Data Fusion | Primary empirical study in narrative synthesis | Used for modality- or commodity-specific findings; no broad transfer or deployment claim was assigned unless independent or online evidence was explicitly reported. |

| | | | | |
|---|---|---|---|---|
| [54] | 2025 | Multi-sensor fusion and deep learning for batch monitoring and real-time warning of apple spoilage | Primary empirical study in narrative synthesis | Used for modality- or commodity-specific findings; no broad transfer or deployment claim was assigned unless independent or online evidence was explicitly reported. |
| [31] | 2024 | Internal quality evaluation of 'Fuji' apples during storage based on bulk optical properties or diffuse reflection and transmission spectra | Primary empirical study in narrative synthesis | Used for modality- or commodity-specific findings; no broad transfer or deployment claim was assigned unless independent or online evidence was explicitly reported. |
| [55] | 2026 | Kolmogorov-Arnold Network-Enhanced Temporal Models on Cloud-Edge System for Real-Time Apple Spoilage Monitoring | Primary empirical study in narrative synthesis | Used for modality- or commodity-specific findings; no broad transfer or deployment claim was assigned unless independent or online evidence was explicitly reported. |
| [58] | 2019 | Automatic grading of apples based on multi-features and weighted K-means clustering algorithm | Primary empirical study in narrative synthesis | Used for modality- or commodity-specific findings; no broad transfer or deployment claim was assigned unless independent or online evidence was explicitly reported. |
| [62] | 2021 | Support vector machine and YOLO for a mobile food grading system | Primary empirical study in narrative synthesis | Used for modality- or commodity-specific findings; no broad transfer or deployment claim was assigned unless independent or online evidence was explicitly reported. |
| [59] | 2021 | Identification of Lycium barbarum varieties based on hyperspectral imaging technique and competitive adaptive reweighted sampling-whale optimization algorithm-support vector machine | Primary empirical study in narrative synthesis | Used for modality- or commodity-specific findings; no broad transfer or deployment claim was assigned unless independent or online evidence was explicitly reported. |
| [63] | 2022 | A comparative analysis of hybrid SVM and LS-SVM classification algorithms to identify dried wolfberry fruits quality based on hyperspectral imaging technology | Primary empirical study in narrative synthesis | Used for modality- or commodity-specific findings; no broad transfer or deployment claim was assigned unless independent or online evidence was explicitly reported. |

| | | | | |
|---|---|---|---|---|
| [64] | 2023 | Nondestructive detection of adulterated wolfberry (Lycium Chinense) fruits based on hyperspectral imaging technology | Primary empirical study in narrative synthesis | Used for modality- or commodity-specific findings; no broad transfer or deployment claim was assigned unless independent or online evidence was explicitly reported. |
| [56] | 2025 | Detection of Early Damage in Kiwifruit Based on Near-Infrared Technology | Primary empirical study in narrative synthesis | Used for modality- or commodity-specific findings; no broad transfer or deployment claim was assigned unless independent or online evidence was explicitly reported. |
| [60] | 2025 | Non-Destructive Detection of Pomegranate Blackheart Disease via Near-Infrared Spectroscopy and Soft X-ray Imaging Systems | Primary empirical study in narrative synthesis | Used for modality- or commodity-specific findings; no broad transfer or deployment claim was assigned unless independent or online evidence was explicitly reported. |
| [61] | 2026 | Quality evaluation and variety discrimination of pear, apple, nectarine and potato using optical properties coupled with machine learning | Primary empirical study in narrative synthesis | Used for modality- or commodity-specific findings; no broad transfer or deployment claim was assigned unless independent or online evidence was explicitly reported. |
| [57] | 2021 | Developing deep learning based regression approaches for prediction of firmness and pH in Kyoho grape using Vis/NIR hyperspectral imaging | Primary empirical study in narrative synthesis | Used for modality- or commodity-specific findings; no broad transfer or deployment claim was assigned unless independent or online evidence was explicitly reported. |
| [65] | 2025 | Hyperspectral Imaging and Deep Learning for Quality and Safety Inspection of Fruits and Vegetables: A Review | Secondary review or contextual synthesis | Mapped for context, terminology or prior synthesis; not assigned an empirical maturity score and not used as direct deployment evidence. |
| [66] | 2023 | Detection model transfer of apple soluble solids content based on NIR spectroscopy and deep learning | Primary empirical study in narrative synthesis | Used for modality- or commodity-specific findings; no broad transfer or deployment claim was assigned unless independent or online evidence was explicitly reported. |
| [67] | 2021 | Fused Deep Features-Based Grape Varieties Identification Using Support Vector Machine | Primary empirical study in narrative synthesis | Used for modality- or commodity-specific findings; no broad transfer or deployment claim was assigned unless independent or online evidence was explicitly reported. |

| | | | | |
|---|---|---|---|---|
| [68] | 2024 | Digital assessment of post-harvest Nendran banana for faster grading: CNN-based ripeness classification model | Primary empirical study in narrative synthesis | Used for modality- or commodity-specific findings; no broad transfer or deployment claim was assigned unless independent or online evidence was explicitly reported. |
| [12] | 2022 | Real-Time Grading of Defect Apples Using Semantic Segmentation Combination with a Pruned YOLO V4 Network | Representative primary empirical study | Formally scored in Table S2; used with the synthesis weight permitted by its maturity category and validation scope. |
| [69] | 2024 | A Lightweight and High-Precision Passion Fruit YOLO Detection Model for Deployment in Embedded Devices | Primary empirical study in narrative synthesis | Used for modality- or commodity-specific findings; no broad transfer or deployment claim was assigned unless independent or online evidence was explicitly reported. |
| [70] | 2021 | Easy domain adaptation method for filling the species gap in deep learning-based fruit detection | Primary empirical study in narrative synthesis | Used for modality- or commodity-specific findings; no broad transfer or deployment claim was assigned unless independent or online evidence was explicitly reported. |
| [71] | 2023 | Lightweight tomato real-time detection method based on improved YOLO and mobile deployment | Primary empirical study in narrative synthesis | Used for modality- or commodity-specific findings; no broad transfer or deployment claim was assigned unless independent or online evidence was explicitly reported. |
| [72] | 2024 | YOLOv8-MDN-Tiny: A lightweight model for multi-scale disease detection of postharvest golden passion fruit | Primary empirical study in narrative synthesis | Used for modality- or commodity-specific findings; no broad transfer or deployment claim was assigned unless independent or online evidence was explicitly reported. |
| [73] | 2023 | Improvement of the prediction of a visual apple ripeness index under seasonal variation by NIR spectral model correction | Primary empirical study in narrative synthesis | Used for modality- or commodity-specific findings; no broad transfer or deployment claim was assigned unless independent or online evidence was explicitly reported. |
| [74] | 2025 | Nondestructive Quality Detection of Characteristic Fruits Based on Vis/NIR Spectroscopy: Principles, Systems, and Applications | Secondary review or contextual synthesis | Mapped for context, terminology or prior synthesis; not assigned an empirical maturity score and not used as direct deployment evidence. |

| | | | | |
|---|---|---|---|---|
| [13] | 2023 | Evaluation of a new apple in-field sorting system for fruit singulation, rotation and imaging | Representative primary empirical study | Formally scored in Table S2; used with the synthesis weight permitted by its maturity category and validation scope. |
| [75] | 2021 | Application of multi-frequency power ultrasound in selected food processing using large-scale reactors: A review | Secondary review or contextual synthesis | Mapped for context, terminology or prior synthesis; not assigned an empirical maturity score and not used as direct deployment evidence. |
| [76] | 2022 | Use of Ultrasonic Cleaning Technology in the Whole Process of Fruit and Vegetable Processing | Secondary review or contextual synthesis | Mapped for context, terminology or prior synthesis; not assigned an empirical maturity score and not used as direct deployment evidence. |
| [78] | 2021 | Experimental Research on Grape Cluster Vibration Signals during Transportation and Placing for Harvest and Post-Harvest Handling | Contextual engineering or handling study | Used for mechanism or equipment-design context; not treated as direct evidence of grading-model transferability or commercial readiness. |
| [79] | 2021 | Hanging force analysis for realizing low vibration of grape clusters during speedy robotic post-harvest handling | Contextual engineering or handling study | Used for mechanism or equipment-design context; not treated as direct evidence of grading-model transferability or commercial readiness. |
| [77] | 2024 | Recent Advances in Light Penetration Depth for Postharvest Quality Evaluation of Fruits and Vegetables | Secondary review or contextual synthesis | Mapped for context, terminology or prior synthesis; not assigned an empirical maturity score and not used as direct deployment evidence. |
| [81] | 2025 | Online assessment of soluble solids content in strawberries using a developed Vis/NIR spectroscopy system with a hanging grasper | Primary empirical study in narrative synthesis | Used for modality- or commodity-specific findings; no broad transfer or deployment claim was assigned unless independent or online evidence was explicitly reported. |
| [14] | 2021 | A Deep Learning-Based Vision System Combining Detection and Tracking for Fast On-Line Citrus Sorting | Representative primary empirical study | Formally scored in Table S2; used with the synthesis weight permitted by its maturity category and validation scope. |

| | | | | |
|---|---|---|---|---|
| [15] | 2024 | A grader for fresh jujube with electromagnetic synchronous actuator assembly | Representative primary empirical study | Formally scored in Table S2; used with the synthesis weight permitted by its maturity category and validation scope. |
| [80] | 2022 | A Soft Gripper Design for Apple Harvesting with Force Feedback and Fruit Slip Detection | Contextual engineering or handling study | Used for mechanism or equipment-design context; not treated as direct evidence of grading-model transferability or commercial readiness. |
| [82] | 2024 | Influence of the peel on online detecting soluble solids content of pomelo using Vis-NIR spectroscopy coupled with chemometric analysis | Primary empirical study in narrative synthesis | Used for modality- or commodity-specific findings; no broad transfer or deployment claim was assigned unless independent or online evidence was explicitly reported. |
| [83] | 2024 | State-of-the-Art Techniques for Fruit Maturity Detection | Secondary review or contextual synthesis | Mapped for context, terminology or prior synthesis; not assigned an empirical maturity score and not used as direct deployment evidence. |
| [84] | 2019 | Maturity assessment of tomato fruit based on electrical impedance spectroscopy | Primary empirical study in narrative synthesis | Used for modality- or commodity-specific findings; no broad transfer or deployment claim was assigned unless independent or online evidence was explicitly reported. |
| [85] | 2025 | Overview of Deep Learning and Nondestructive Detection Technology for Quality Assessment of Tomatoes | Secondary review or contextual synthesis | Mapped for context, terminology or prior synthesis; not assigned an empirical maturity score and not used as direct deployment evidence. |
| [86] | 2025 | Advances in Computer Vision and Spectroscopy Techniques for Non-Destructive Quality Assessment of Citrus Fruits: A Comprehensive Review | Secondary review or contextual synthesis | Mapped for context, terminology or prior synthesis; not assigned an empirical maturity score and not used as direct deployment evidence. |
| [87] | 2019 | Monitoring strategies for quality control of agricultural products using visible and near-infrared spectroscopy: A review | Secondary review or contextual synthesis | Mapped for context, terminology or prior synthesis; not assigned an empirical maturity score and not used as direct deployment evidence. |

| | | | | |
|---|---|---|---|---|
| [88] | 2018 | Near infrared spectroscopy: A mature analytical technique with new perspectives - A review | Secondary review or contextual synthesis | Mapped for context, terminology or prior synthesis; not assigned an empirical maturity score and not used as direct deployment evidence. |
| [89] | 2025 | A Computer Vision and AI-Based System for Real-Time Sizing and Grading of Thai Export Fruits | Primary empirical study in narrative synthesis | Used for modality- or commodity-specific findings; no broad transfer or deployment claim was assigned unless independent or online evidence was explicitly reported. |
| [90] | 2025 | Improving SSC detection accuracy of hanging-transported strawberries through different correction methods and 1D-CNN | Primary empirical study in narrative synthesis | Used for modality- or commodity-specific findings; no broad transfer or deployment claim was assigned unless independent or online evidence was explicitly reported. |
| [91] | 2022 | Fuzzy Comprehensive Evaluation for Grasping Prioritization of Stacked Fruit Clusters Based on Relative Hierarchy Factor Set | Contextual engineering or handling study | Used for mechanism or equipment-design context; not treated as direct evidence of grading-model transferability or commercial readiness. |
| [92] | 2025 | Automatic Fruit Grading System with High Adaptability Using Machine Learning Method | Primary empirical study in narrative synthesis | Used for modality- or commodity-specific findings; no broad transfer or deployment claim was assigned unless independent or online evidence was explicitly reported. |
| [93] | 2023 | Designing, Optimizing, and Validating a Low-Cost, Multi-Purpose, Automatic System-Based RGB Color Sensor for Sorting Fruits | Primary empirical study in narrative synthesis | Used for modality- or commodity-specific findings; no broad transfer or deployment claim was assigned unless independent or online evidence was explicitly reported. |
| [94] | 2017 | Grasping damage analysis of apple by end-effector in harvesting robot | Contextual engineering or handling study | Used for mechanism or equipment-design context; not treated as direct evidence of grading-model transferability or commercial readiness. |
| [95] | 2021 | An Octopus-Inspired Bionic Flexible Gripper for Apple Grasping | Contextual engineering or handling study | Used for mechanism or equipment-design context; not treated as direct evidence of grading-model transferability or commercial readiness. |

| | | | | |
|---|---|---|---|---|
| [96] | 2023 | Research on Flexible End-Effectors with Humanoid Grasp Function for Small Spherical Fruit Picking | Contextual engineering or handling study | Used for mechanism or equipment-design context; not treated as direct evidence of grading-model transferability or commercial readiness. |
| [97] | 2021 | Soft Grippers for Automatic Crop Harvesting: A Review | Secondary review or contextual synthesis | Mapped for context, terminology or prior synthesis; not assigned an empirical maturity score and not used as direct deployment evidence. |
| [98] | 2024 | Design and Preliminary Evaluation of Automated Sweetpotato Sorting Mechanisms | Contextual engineering or handling study | Used for mechanism or equipment-design context; not treated as direct evidence of grading-model transferability or commercial readiness. |
| [99] | 2024 | Nondestructive determination of edible quality and watercore degree of apples by portable Vis/NIR transmittance system combined with CARS-CNN | Primary empirical study in narrative synthesis | Used for modality- or commodity-specific findings; no broad transfer or deployment claim was assigned unless independent or online evidence was explicitly reported. |
| [16] | 2024 | Non-destructive online detection of early moldy core apples based on Vis/NIR transmission spectroscopy | Representative primary empirical study | Formally scored in Table S2; used with the synthesis weight permitted by its maturity category and validation scope. |
| [100] | 2023 | Multi-Index Grading Method for Pear Appearance Quality Based on Machine Vision | Primary empirical study in narrative synthesis | Used for modality- or commodity-specific findings; no broad transfer or deployment claim was assigned unless independent or online evidence was explicitly reported. |
| [101] | 2022 | Complementary deep learning and chemometrics: A case of pear fruit centroid detection and spectral model application for fruit spectral image processing | Primary empirical study in narrative synthesis | Used for modality- or commodity-specific findings; no broad transfer or deployment claim was assigned unless independent or online evidence was explicitly reported. |
| [102] | 2024 | Detection of Pear Quality Using Hyperspectral Imaging Technology and Machine Learning Analysis | Primary empirical study in narrative synthesis | Used for modality- or commodity-specific findings; no broad transfer or deployment claim was assigned unless independent or online evidence was explicitly reported. |

| | | | | |
|---|---|---|---|---|
| [103] | 2018 | Postharvest Dry Matter and Soluble Solids Content Prediction in d'Anjou and Bartlett Pear Using Near-infrared Spectroscopy | Primary empirical study in narrative synthesis | Used for modality- or commodity-specific findings; no broad transfer or deployment claim was assigned unless independent or online evidence was explicitly reported. |
| [104] | 2023 | Application of deep learning diagnosis for multiple traits sorting in peach fruit | Primary empirical study in narrative synthesis | Used for modality- or commodity-specific findings; no broad transfer or deployment claim was assigned unless independent or online evidence was explicitly reported. |
| [17] | 2024 | Early Detection of Slight Bruises in Yellow Peaches (Amygdalus persica) Using Multispectral Structured-Illumination Reflectance Imaging and an Improved Otsu Method | Representative primary empirical study | Formally scored in Table S2; used with the synthesis weight permitted by its maturity category and validation scope. |
| [105] | 2018 | Evaluation of plum fruit maturity by image processing techniques | Primary empirical study in narrative synthesis | Used for modality- or commodity-specific findings; no broad transfer or deployment claim was assigned unless independent or online evidence was explicitly reported. |
| [106] | 2025 | Non-Destructive Detection and Grading of Plum Quality Based on Multimodal Data | Primary empirical study in narrative synthesis | Used for modality- or commodity-specific findings; no broad transfer or deployment claim was assigned unless independent or online evidence was explicitly reported. |
| [107] | 2024 | Prediction of prunoideae fruit quality characteristics based on machine learning and spectral characteristic acquisition optimization | Primary empirical study in narrative synthesis | Used for modality- or commodity-specific findings; no broad transfer or deployment claim was assigned unless independent or online evidence was explicitly reported. |
| [18] | 2025 | Vis/NIR Spectroscopy and Vis/NIR Hyperspectral Imaging for Non-Destructive Monitoring of Apricot Fruit Internal Quality with Machine Learning | Representative primary empirical study | Formally scored in Table S2; used with the synthesis weight permitted by its maturity category and validation scope. |

| | | | | |
|---|---|---|---|---|
| [108] | 2022 | Hyperspectral dimension reduction and navel orange surface disease defect classification using independent component analysis-genetic algorithm | Primary empirical study in narrative synthesis | Used for modality- or commodity-specific findings; no broad transfer or deployment claim was assigned unless independent or online evidence was explicitly reported. |
| [109] | 2024 | Fast detection of the early decay in oranges using visible-LED structured- illumination imaging combined with spiral phase transform and feature-based classification model | Primary empirical study in narrative synthesis | Used for modality- or commodity-specific findings; no broad transfer or deployment claim was assigned unless independent or online evidence was explicitly reported. |
| [110] | 2024 | Qualitative and quantitative analysis of Nanfeng mandarin quality based on hyperspectral imaging and deep learning | Primary empirical study in narrative synthesis | Used for modality- or commodity-specific findings; no broad transfer or deployment claim was assigned unless independent or online evidence was explicitly reported. |
| [111] | 2025 | External defect detection of Orah mandarin based on a non-brightness correction algorithm | Primary empirical study in narrative synthesis | Used for modality- or commodity-specific findings; no broad transfer or deployment claim was assigned unless independent or online evidence was explicitly reported. |
| [19] | 2023 | Detection and Classification of Citrus Fruit Infestation by Bactrocera dorsalis (Hendel) Using a Multi-Path Vis/NIR Spectroscopy System | Representative primary empirical study | Formally scored in Table S2; used with the synthesis weight permitted by its maturity category and validation scope. |
| [112] | 2023 | Characterisation and optical detection of puffy Satsuma mandarin | Primary empirical study in narrative synthesis | Used for modality- or commodity-specific findings; no broad transfer or deployment claim was assigned unless independent or online evidence was explicitly reported. |
| [113] | 2024 | Prediction of Soluble-Solid Content in Citrus Fruit Using Visible-Near-Infrared Hyperspectral Imaging Based on Effective-Wavelength Selection Algorithm | Primary empirical study in narrative synthesis | Used for modality- or commodity-specific findings; no broad transfer or deployment claim was assigned unless independent or online evidence was explicitly reported. |

| | | | | |
|---|---|---|---|---|
| [114] | 2025 | Evaluating ripeness in post-harvest stored kiwifruit using VIS-NIR hyperspectral imaging | Primary empirical study in narrative synthesis | Used for modality- or commodity-specific findings; no broad transfer or deployment claim was assigned unless independent or online evidence was explicitly reported. |
| [115] | 2020 | Kinetic models applied to quality change and shelf life prediction of kiwifruits | Primary empirical study in narrative synthesis | Used for modality- or commodity-specific findings; no broad transfer or deployment claim was assigned unless independent or online evidence was explicitly reported. |
| [116] | 2024 | Non-destructive prediction of ready-to-eat kiwifruit firmness based on Fourier transform near-infrared spectroscopy | Primary empirical study in narrative synthesis | Used for modality- or commodity-specific findings; no broad transfer or deployment claim was assigned unless independent or online evidence was explicitly reported. |
| [117] | 2021 | Mango maturity classification instead of maturity index estimation: A new approach towards handheld NIR spectroscopy | Primary empirical study in narrative synthesis | Used for modality- or commodity-specific findings; no broad transfer or deployment claim was assigned unless independent or online evidence was explicitly reported. |
| [118] | 2024 | Non-destructive methods for mango ripening prediction: Visible and near-infrared spectroscopy (visNIRS) and laser Doppler vibrometry (LDV) | Primary empirical study in narrative synthesis | Used for modality- or commodity-specific findings; no broad transfer or deployment claim was assigned unless independent or online evidence was explicitly reported. |
| [119] | 2018 | Integration of computer vision and colorimetric sensor array for nondestructive detection of mango quality | Primary empirical study in narrative synthesis | Used for modality- or commodity-specific findings; no broad transfer or deployment claim was assigned unless independent or online evidence was explicitly reported. |
| [120] | 2023 | Nondestructive Detecting Maturity of Pineapples Based on Visible and Near-Infrared Transmittance Spectroscopy Coupled with Machine Learning Methodologies | Primary empirical study in narrative synthesis | Used for modality- or commodity-specific findings; no broad transfer or deployment claim was assigned unless independent or online evidence was explicitly reported. |

| | | | | |
|---|---|---|---|---|
| [121] | 2025 | Surface Defect and Malformation Characteristics Detection for Fresh Sweet Cherries Based on YOLOv8-DCPF Method | Primary empirical study in narrative synthesis | Used for modality- or commodity-specific findings; no broad transfer or deployment claim was assigned unless independent or online evidence was explicitly reported. |
| [20] | 2025 | Maturity Classification of Blueberry Fruit Using YOLO and Vision Transformer for Agricultural Assistance | Representative primary empirical study | Formally scored in Table S2; used with the synthesis weight permitted by its maturity category and validation scope. |
| [122] | 2025 | Non-destructive early bruise detection of blueberries based on hyperspectral image feature fusion PKOA-SVM model | Primary empirical study in narrative synthesis | Used for modality- or commodity-specific findings; no broad transfer or deployment claim was assigned unless independent or online evidence was explicitly reported. |
| [21] | 2019 | Measuring Internal Maturity Parameters Contactless on Intact Table Grape Bunches Using NIR Spectroscopy | Representative primary empirical study | Formally scored in Table S2; used with the synthesis weight permitted by its maturity category and validation scope. |
| [123] | 2026 | Study on Quality Detection Methods for Table Grapes Based on Spectral and Imaging Information | Primary empirical study in narrative synthesis | Used for modality- or commodity-specific findings; no broad transfer or deployment claim was assigned unless independent or online evidence was explicitly reported. |
